# Modelling African Swine Fever Transmission and Epidemiology: A Scoping Review of Mechanistic, Statistical, and Machine Learning Approaches

**DOI:** 10.3390/tropicalmed11080228

**Published:** 2026-08-14

**Authors:** Kim Dianne B. Ligue-Sabio, Yoni Nazarathy, Kien Quoc Do, Luis Furuya-Kanamori, Yusuf A. Sucol, Benn Sartorius, Colleen L. Lau

**Affiliations:** 1 Frazer Institute, Faculty of Health, Medicine and Behavioural Sciences, The University of Queensland, Brisbane, QLD 4006, Australia; 2 Department of Mathematics, Physics, and Computer Science, College of Science and Mathematics, University of the Philippines Mindanao, Davao City 8022, Philippines; 3 School of Mathematics and Physics, Faculty of Science, The University of Queensland, Brisbane, QLD 4072, Australia; 4 School of Public Health, Faculty of Health, Medicine and Behavioural Sciences, The University of Queensland, Brisbane, QLD 4006, Australia; kienquoc.do@uq.edu.au; 5 UPLB Climate and Disaster Risks Studies Center, School of Environmental Science and Management, University of the Philippines Los Baños, Los Baños 4031, Philippines; yasucol@up.edu.ph

**Keywords:** infectious disease modelling, transboundary animal disease, veterinary epidemiology, disease surveillance, uncertainty quantification

## Abstract

African swine fever (ASF) is a viral disease of domestic and wild pigs that has re-emerged as a major transboundary disease. Modelling using mechanistic, statistical, and machine learning (ML) approaches plays a key role in understanding ASF transmission and informing disease control, but the literature remains fragmented. To synthesise global ASF modelling efforts, we systematically reviewed studies applying these three approaches. We examined temporal and geographic trends, modelling objectives, explanatory variables, and model evaluation practices. A total of 151 papers published through 2024 met the inclusion criteria. Mechanistic (54.3%) and statistical (40.4%) approaches predominated, whereas ML (9.3%) was increasingly applied in recent years. Mechanistic models were primarily used to assess control strategies (48.8%) and transmission drivers (41.5%), statistical models to identify risk factors (63.9%) and spatiotemporal spread (32.8%), and ML for environmental suitability modelling (64.3%). Most were published from 2011 (99.3%) and focused on Europe (43.0%) and Asia (26.5%). Model evaluation remained inconsistent, with mechanistic papers frequently lacking model output uncertainty quantification (47.0%) and statistical papers often omitting model adequacy assessment (49.2%) and assumption checking (50.8%). Overall, ASF modelling approaches have developed complementary methodological roles, while geographic underrepresentation, limited representation of some transmission pathways, and inconsistent model evaluation remain important gaps.

## 1. Introduction

African swine fever (ASF) is a viral disease affecting domestic pigs and wild boars, with case fatality rates approaching 100% for highly virulent strains. It is caused by the African swine fever virus (ASFV), a large double-stranded DNA virus with high genetic variability [1]. First detected in sub-Saharan Africa in the early 1900s, ASF spread outside Africa through multiple introductions, beginning with genotype I incursions into Europe in 1957 and 1960, followed by the introduction of ASFV genotype II into Georgia in 2007. Since then, ASFV genotype II has driven the current transcontinental spread across Europe, Asia, Oceania, and the reappearance of ASF in the Americas, resulting in widespread outbreaks and severe socioeconomic and food security impacts across multiple continents [2]. With mortality rates approaching 100% and an exceptional capacity for rapid intercontinental spread, ASF is recognised as one of the most important transboundary animal diseases (TADs), posing a major threat to global swine health [3].

The epidemiology of ASF is shaped by the combined effects of multi-host ecology, environmental persistence, and anthropogenic factors [2]. The ASF virus circulates among domestic pigs and wild boar via direct pig-to-pig transmission. In some regions, vector-borne transmission also occurs via soft ticks (e.g., the *Ornithodoros* tick cycle), such as in sub-Saharan Africa where the warthog–tick sylvatic cycle remains endemic [4]. ASFV has exceptional environmental stability, allowing it to persist for extended periods in carcasses, meat products, and fomites, facilitating both local persistence and long-distance spread [5]. Furthermore, the epidemiological context varies widely across regions, influenced by factors such as wild boar density, pig farming systems, and the implementation of biosecurity and control measures [6]. These interacting biological and socioeconomic drivers create highly heterogeneous outbreak dynamics that are difficult to understand and control.

Modelling provides a systematic framework to untangle this complexity and inform prevention and control strategies [7]. A diverse range of methods has been applied to modelling ASF epidemiology and transmission dynamics, with three broad approaches emerging as central to disease modelling: mechanistic, statistical, and machine learning (ML) [8]. These modelling frameworks offer distinct methodological lenses and representations of disease dynamics, differing in underlying structural assumptions. Mechanistic models aim to capture the biological and epidemiological processes driving disease transmission by specifying how system states evolve through mathematical equations or rule-based simulations, making them particularly useful for exploring disease dynamics and testing hypothetical control strategies [9]. Statistical models, by contrast, use pre-specified statistical structures to quantify associations between disease outcomes and explanatory variables, with data informing parameter estimates within that structure, supporting inference, hypothesis testing, and uncertainty quantification [10]. More recently, ML approaches have gained traction as powerful data-driven tools that learn functional relationships directly from data while imposing relatively few assumptions about underlying biological processes or statistical relationships, thereby offering new opportunities for prediction and real-time decision support [11]. Although these approaches are presented as distinct categories, they should not be regarded as mutually exclusive. Rather, the taxonomy adopted in this review reflects differences in the extent to which model structure is specified a priori or learned from data and is intended as a pragmatic framework for organising the evidence, rather than as a rigid classification.

Interest in ASF modelling has grown considerably in recent years. Following the re-emergence of ASFV in Georgia in 2007 and its subsequent spread across Europe and Asia, ASF modelling has received increasing research attention. A 2021 review of mechanistic ASF models identified 34 publications from 2011 to 2020, with a sharp increase in 2020 alone, which accounted for nearly one-third of the studies reviewed [9]. In 2021, the ASF Modelling Challenge was also launched, marking the first global modelling competition focused on an animal disease [12], further demonstrating the growing momentum in this field. Following the surge in publications in 2020, research output is expected to continue to increase. However, a 2022 review on ML and deep learning for disease prediction revealed that non-zoonotic animal diseases like ASF remain significantly underrepresented compared to human and zoonotic diseases such as COVID-19 and avian influenza [8]. While recent developments signal important progress, the literature remains fragmented, with limited synthesis across modelling approaches, resulting in gaps in understanding about how different frameworks contribute to ASF research and control efforts. This highlights a critical gap in the broader infectious disease modelling literature and underscores the need for a dedicated review to synthesise existing ASF modelling efforts and assess how they have been applied to advance understanding of disease dynamics, control, and prevention.

In light of these gaps, this study aims to summarise the evolution and application of ASF modelling using mechanistic, statistical, and ML approaches. To achieve this aim, we conducted a scoping review of modelling papers published as journal articles to identify studies that applied these approaches to model ASF transmission dynamics and epidemiology in pigs. By mapping existing modelling strategies, this review seeks to inform future research directions and support the development of context-appropriate models, thereby supporting future modelling efforts that underpin ASF surveillance, control, and response.

## 2. Materials and Methods

### 2.1. Research Protocol

We conducted a scoping review reported in accordance with the Preferred Reporting Items for Systematic Reviews and Meta-Analyses (PRISMA) guidelines for Scoping Reviews [13] (Supplementary Material S1A). Specifically, this review was guided by the following research questions:

1. What modelling objectives have been addressed by each approach?
2. How have patterns of use of these modelling techniques evolved over time and across geographic regions?
3. What types of ASF data have been modelled, and which factors have been considered as explanatory variables or input features in these models?
4. How was model performance evaluated?

The research protocol was registered in International Platform of Registered Systematic Review and Meta-analysis Protocols (INPLASY) under the registration number INPLASY2025100034 (available at https://inplasy.com/inplasy-2025-10-0034/, accessed on 30 July 2026).

### 2.2. Eligibility Criteria

Papers were selected based on predefined eligibility criteria. We included papers that explicitly applied mechanistic, statistical, or ML approaches to model ASF and were published in English as journal articles up to 31 December 2024. Papers were eligible if they modelled ASF-related outcomes as the response variable, such as presence or absence of ASFV, or counts of infected units from outbreak notifications or surveillance data. These data types could be reported at the level of individual animals, herds/farms, or aggregated administrative units. We excluded papers that modelled biomarker data (e.g., genomics, proteomics, transcriptomics) or non-ASF data (e.g., economic indicators, knowledge–attitude–perception (KAP) responses, farm biosecurity assessments, trade movements). Papers were also excluded if they applied statistical techniques solely for hypothesis testing (e.g., ANOVA/Kruskal–Wallis tests, Pearson/Spearman correlations, Chi-square/Fisher’s tests for association, Global Moran’s I). We excluded preprints, conference proceedings, reviews, book chapters, and dissertations, as well as articles with missing or inaccessible full texts.

### 2.3. Search Strategy and Data Source

A comprehensive search strategy was developed using keywords associated with four main topics: ASF, mechanistic models, statistical models, and machine learning. Searches were conducted in PubMed, Embase, Web of Science, and Scopus on 22 November 2024 and repeated on 1 August 2025 to capture all relevant papers published up to 31 December 2024. Search terms were applied to the title, abstract, and keyword fields, and controlled vocabulary was used where applicable, i.e., Medical Subject Headings (MeSH) in PubMed and Emtree in Embase, while indexed terms were applied for Scopus. Additional papers were identified by hand-searching the reference lists of included articles. The full search strategies for each database are provided in Supplementary Material S1B.

### 2.4. Study Selection

Screening and selection were conducted using Covidence [14]. After automated duplicate removal, two researchers (KDLS and KQD) independently screened titles and abstracts to identify potentially eligible papers and reviewed full texts against the inclusion criteria. Disagreements were resolved by a third independent reviewer (BS or YN), depending on subject-matter expertise. Included papers and their full texts were then managed in Endnote 21 for extraction [15].

### 2.5. Data Extraction and Synthesis

Data extracted from each publication included modelling approach, objectives, methods, study location, publication year, model structure, data inputs, model adequacy, and uncertainty quantification. For mechanistic models, following the framework of Hayes et al. (2021) [9], we further extracted model descriptors (basic epidemiological unit and transmission cycle), model components including framework (population-based, individual-based, or metapopulation), approaches to uncertainty (deterministic or stochastic), time (discrete or continuous), space (spatially explicit, accounting for movement, distance, or both, or non-spatially explicit), and uncertainty quantification. For statistical and ML models, following the framework of Keshavamurthy et al. (2022) [8], we collected details on explanatory variables/input features, assumptions testing, model adequacy checks, and uncertainty quantification. Model adequacy checks included assessments of model fit, predictive performance, explanatory power, validation procedures, and general model diagnostics, as appropriate to the method used. Some papers used multiple approaches and/or models. In such cases, information was extracted for each model using the corresponding framework for that approach. However, results were reported at the paper level. Therefore, categories are not mutually exclusive, and a single paper may contribute to multiple categories. Standard risk of bias assessments (e.g., RoB 2, ROBINS-I) were not applicable and were therefore not employed given the modelling focus of this review. Data extraction was performed by KDLS and reviewed by KQD, with disagreements resolved through internal discussion until consensus was achieved.

### 2.6. Clustering Analysis

To quantitatively characterise associations between modelling approach, objective, publication year, and continent of study location, we applied Hierarchical Clustering on Principal Components (HCPC). Individual model implementations were treated as the unit of analysis, with papers applying multiple modelling approaches contributing one observation for each model implementation. We used the ‘FactoMineR’ package in R version 4.4.0 because it allowed us to handle mixed data. HCPC first embedded observations in a low-dimensional factor space using Factor Analysis of Mixed Data (FAMD), accommodating both numeric (year) and categorical (approach, objective, continent) variables. Ward’s $D^{2}$ hierarchical clustering was then computed on the factor coordinates, and the number of clusters was determined by the automated HCPC criterion, with optional refinement by *k*-means consolidation. Over- and underrepresented category levels per cluster were identified using the *v*-test statistic, where positive and negative values indicated over- and under-representation, respectively. To aid interpretation, we also reported Mod/Cla (proportion of papers in the cluster exhibiting the category), Cla/Mod (proportion of papers with the category that belong to the cluster), and global values (overall prevalence in the dataset). A supplementary Kruskal–Wallis test was performed to assess whether publication year differed significantly among clusters. Statistical significance was assessed at *p* < 0.05 for all statistical tests. The R code used for the HCPC analysis and Kruskal–Wallis test is provided in Supplementary Material S1H.

## 3. Results

**Figure 1:**
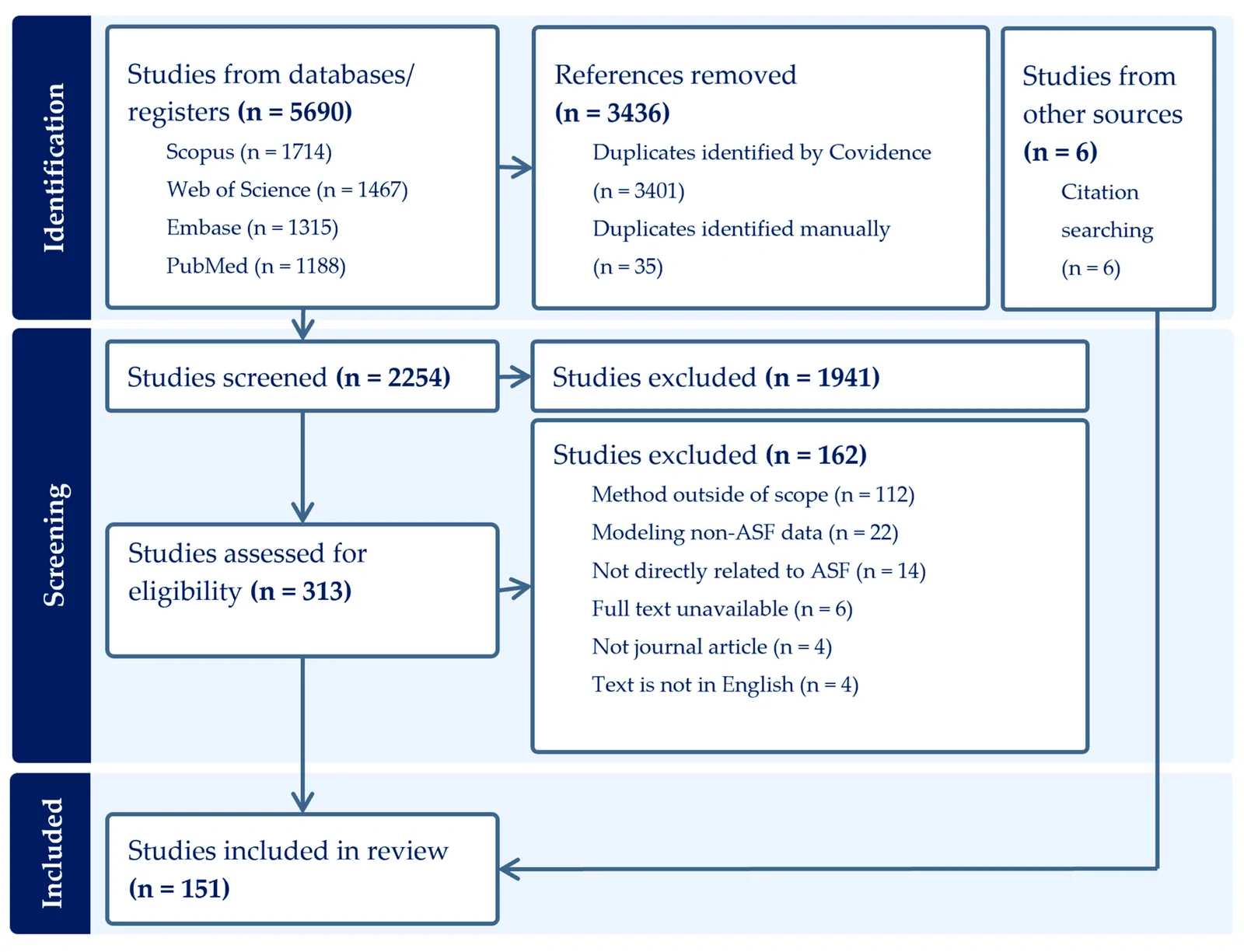
Preferred Reporting Items for Systematic Reviews and Meta-Analyses extension for Scoping Reviews (PRISMA-ScR) flow diagram illustrating the overall selection process.

Our search identified 5690 papers across four databases (Figure 1). After deduplication and screening based on eligibility criteria, 151 papers (2.7%) were included in the review. Of these, six papers were identified through manual screening of the reference lists of included papers. The complete list of included papers and extracted data are provided in Supplementary Material S2.

### 3.1. Trend Across Time and Geographic Focus

**Figure 2:**
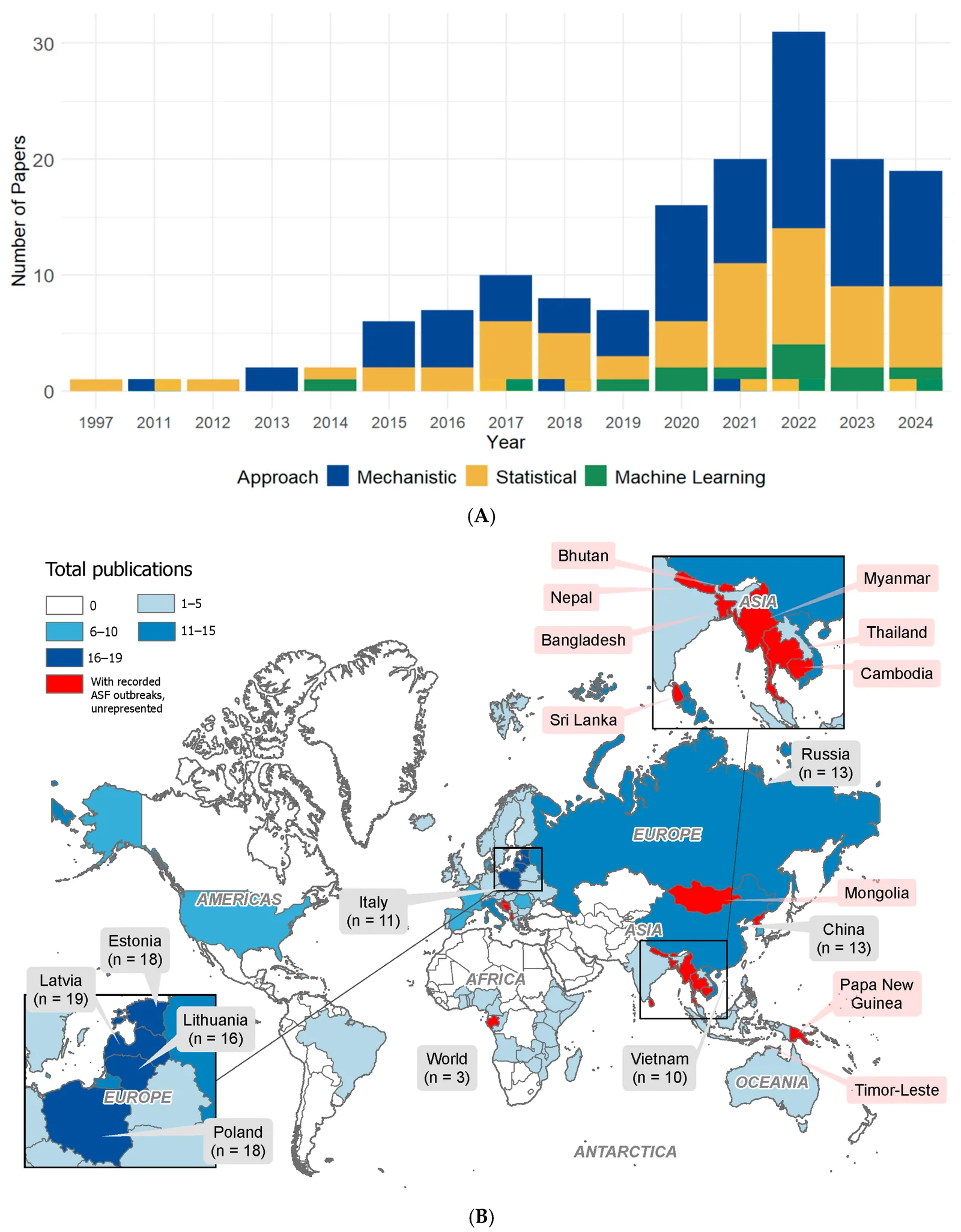
Distribution of African swine fever modelling papers (1997–2024): (**A**) by publication year, and (**B**) by geographic distribution. In (**A**), horizontally split colour segments denote papers applying more than one approach. In (**B**), grey and red labels indicate represented and unrepresented countries in the ASF modelling literature, respectively.

Among the 151 papers, ASF modelling papers primarily employed mechanistic (82, 54.3%) and statistical (61, 40.4%) approaches, while only a few applied ML (14, 9.3%). Six papers (4.0%) applied more than one approach to address distinct modelling objectives. All but one were published from 2011, with a notable increase from 2020 onwards (Figure 2A). The majority examined ASF spread in Europe (65, 43.0%), Asia (40, 26.5%), and Africa (11, 7.3%), while fewer focused on the Americas (8, 5.3%) and Oceania (1, 0.7%). Additionally, three ML papers (2.0%) considered ASF at the global scale, whereas 23 mechanistic papers (15.2%) explored simulated scenarios in non-specific or fictional settings (Supplementary Material S1C). At the country level, most papers in Europe focused on the Baltic states, namely Latvia (19, 12.6%), Estonia (18, 11.9%), and Lithuania (16, 10.6%), and Poland (18, 11.9%), while Asia, China (13, 8.6%) and Vietnam (10, 6.6%) produced the largest number of publications (Figure 2B).

**Figure 3:**
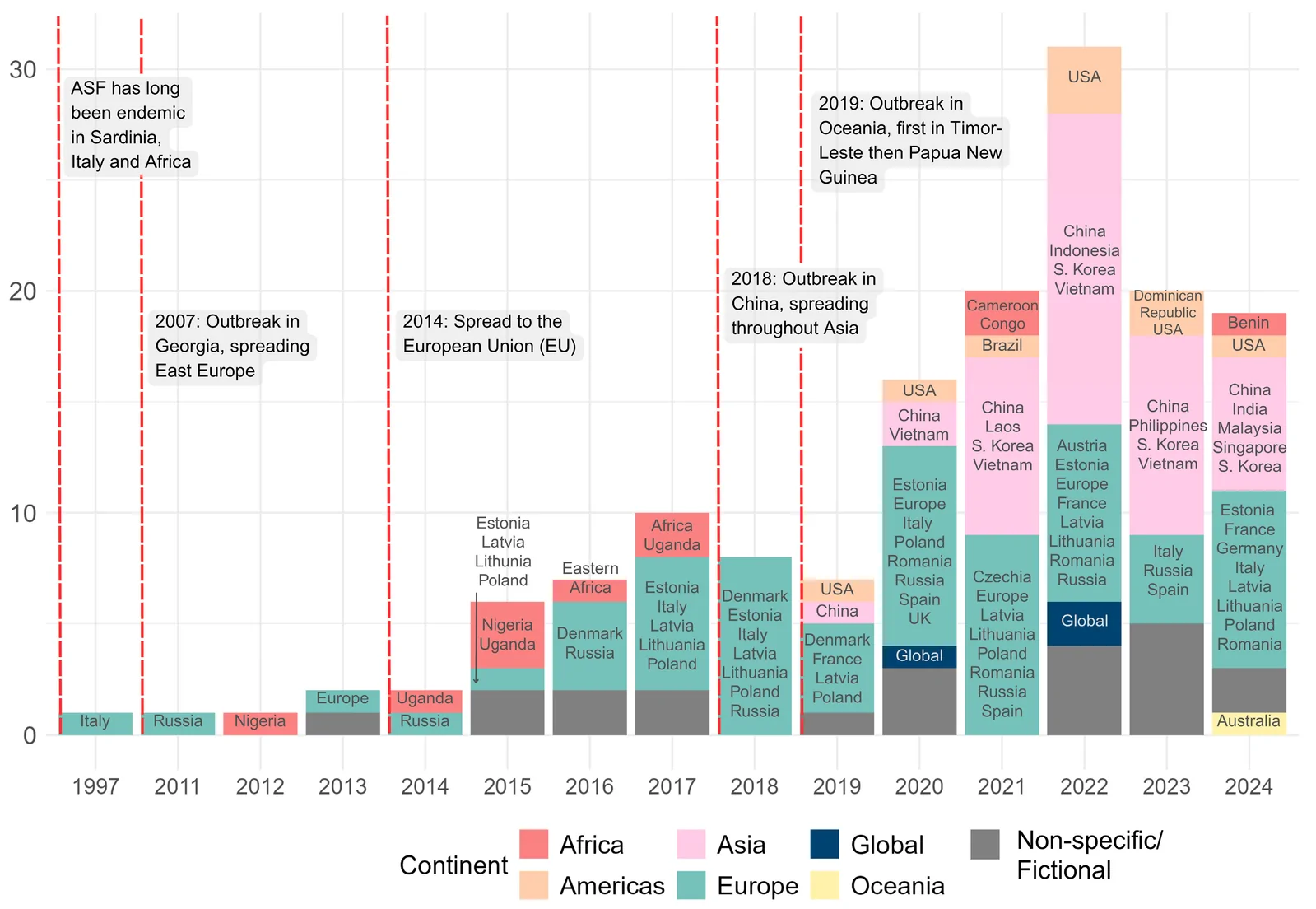
Distribution of African swine fever (ASF) modelling papers by publication year (1997–2024) and geographic focus, mapped against notable ASF events (red dashed lines). Bars are coloured by continent and labelled by specific countries of study location.

Mapping key ASF outbreaks against publication timelines and study locations reveals patterns in research focus mirroring global ASF spread (Figure 3). With ASF long endemic in Africa and Sardinia, Italy, and the 2007 outbreak in Georgia leading to the subsequent spread throughout Europe, research efforts were largely limited to Europe and Africa until 2018. Following ASF introduction to the European Union (EU) in 2014, a rise in the number of scientific publications was observed. By 2018, ASF reached China and rapidly spread throughout Asia, leading to an increase in studies focused on Asian countries from 2019 onwards. In response to the intercontinental spread and re-emergence in some regions, countries in the Americas and Australia began exploring hypothetical scenarios to prepare for potential outbreaks. Throughout this period, several studies also examined simulated scenarios in non-specific or fictional settings.

The steady increase in the number of publications reflects intensified global research activity in response to the expanding epidemic. Nevertheless, several affected countries remain underrepresented in the literature. For instance, ASF has spread within Oceania since 2019, particularly in Timor-Leste and Papua New Guinea, yet ASF modelling research focusing on these countries has yet to be documented. Similarly, several Asian countries have experienced ASF outbreaks but remain unrepresented, including Bangladesh, Bhutan, Cambodia, the Democratic People’s Republic of Korea, Mongolia, Myanmar, Nepal, Thailand, and Sri Lanka (Figure 2B).

Mechanistic and statistical model applications have shown parallel growth since 2011, contrasted by the recent introduction of ML approaches from 2014 (Supplementary Material S1D). Mechanistic modelling expanded substantially following the previous review by Hayes et al. [9], with the number of published studies increasing by approximately 1.4-fold between 2021 and 2024. Among 82 mechanistic modelling papers, compartmental (53, 64.6%) and agent-based models (27, 32.9%) were most frequently applied throughout the years, with network models (11, 13.4%) slowly rising in use from 2019. Among 61 statistical modelling papers, regression-based generalised linear models (GLMs) and extensions (47, 77.0%) were most common, followed by space–time clustering using Kulldorf’s scan statistic (17, 27.9%) which gained popularity from 2016. ML-based modelling was sparse and slowly gained traction in use recently, with the majority (12, 85.7%) of the 14 papers published from 2018. All ML papers employed supervised ML, with one paper also incorporating an unsupervised space–time clustering technique in combination with (supervised) regression analysis [16]. Among the supervised ML papers, maximum entropy (MaxEnt) model was the most common (9, 64.3%), while the remaining papers employed tree-based and ensemble methods.

### 3.2. Modelling Approach and Objectives

**Figure 4:**
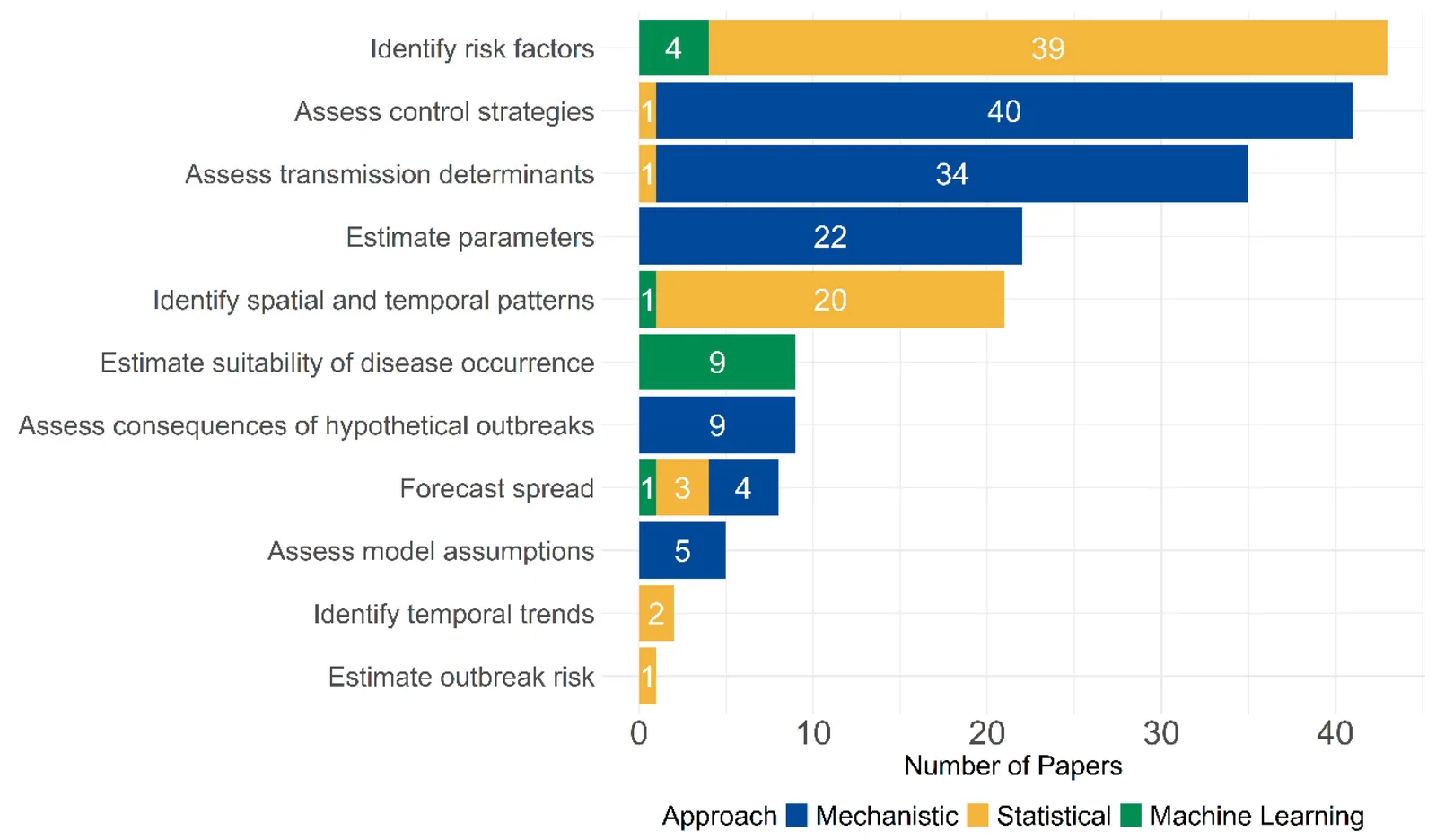
Distribution of African swine fever modelling papers by modelling objectives and approach. Some papers employed multiple approaches and/or models, and some models addressed more than one objective. We distinguished between ‘risk factors’ and ‘transmission determinants’ to maintain important differences in methodological intentions across papers. Risk factors refer to variables associated with models estimating the likelihood of ASF occurrence, while transmission determinants describe factors influencing how the disease spreads.

Across the 151 papers, modelling objectives were grouped into 11 distinct categories (Figure 4). Different modelling approaches predominated across modelling objectives, with some objectives addressed using multiple approaches.

Mechanistic models were mainly employed to assess control strategies (40, 48.8%) [17,18,19,20,21,22,23,24,25,26,27,28,29,30,31,32,33,34,35,36,37,38,39,40,41,42,43,44,45,46,47,48,49,50,51,52,53,54,55,56] and transmission determinants (34, 41.5%) [19,24,26,28,29,30,32,33,34,43,46,51,53,57,58,59,60,61,62,63,64,65,66,67,68,69,70,71,72,73,74,75,76,77]. Unique to this modelling approach was parameter estimation, primarily of $R_{0}$, transmission rates, and durations between infection states (22, 26.8%) [23,29,44,71,73,78,79,80,81,82,83,84,85,86,87,88,89,90,91,92,93,94], and evaluation of hypothetical outbreak scenarios (9, 11.0%) [25,40,41,42,54,57,58,77,95]. Notably, about 60% of these mechanistic models addressed multiple objectives within a single framework. Recent models incorporated a broader range of epidemiological processes, including environmental persistence, arthropod vectors, human behavioural factors, and transmission at the wildlife–livestock interface. Six studies simulated transmission between domestic pigs and wild boar [20,28,35,52,70,94], three of which were developed for the 2021 ASF Modelling Challenge [20,35,52]. Wild boar modelling also expanded beyond its earlier European focus to include studies in Asia [37,48,89] and simulated outbreaks in Australia [77] and the United States [45]. Among model structures, stochastic metapopulation frameworks were the most frequently applied [17,18,20,22,27,29,34,35,36,38,40,41,42,45,46,50,52,53,54,56,59,60,65,67,72,77,81,82,83,84,85,86,89].

When assessing control strategies, compartmental models were applied globally across diverse contexts. In Europe, they were widely used to evaluate policy interventions centred on culling, surveillance, and movement restrictions in domestic pigs [22,38,40,54]. Several studies also assessed the effectiveness of biosecurity measures in pig production systems in Asia [19,23,30,39,44] and in non-specific settings [24,28,33,55]. Agent-based models have been mostly applied to study wild boar control strategies, including carcass removal and population reduction measures, largely in Europe [18,21,25,27,32,41,46,50,56] and in Asia [37,48]. These models allowed explicit representation of individual movement, spatial behaviour, and social structure, which are critical for free-roaming wildlife populations. By contrast, network models have been used to incorporate animal movements in domestic pig systems in the Americas [29,34,42] and Europe [40,54], enabling the evaluation of movement-based control strategies.

Among mechanistic studies assessing transmission determinants, the pathways represented varied according to host system and geographic setting. Animal movement and trade networks were commonly modelled in domestic pig production systems globally using network models [29,34,65,67] or compartmental models with metapopulation frameworks [53,59]. Asian studies also frequently incorporated farm-level biosecurity practices, particularly swill feeding [30,65,71]. In contrast, studies involving wild boar, predominantly conducted in Europe, commonly examined host density, population structure, and carcass-mediated transmission across a range of mechanistic modelling frameworks [26,43,61,62,64,68,72,75,76,77]. Outside Europe, wild boar was represented only in studies from South Korea [96,97] and Singapore [37]. Vector-borne transmission via ticks and stable flies was evaluated only in hypothetical outbreak scenarios using compartmental models [19,28,33,63,70], where vectors were represented as separate compartments transmitting infection between susceptible and infectious hosts. Although ticks were acknowledged in the epidemiological background of all three African mechanistic studies [36,74,80], they were not explicitly incorporated into model structure.

Estimates of $R_{0}$ varied according to the transmission cycle examined (Supplementary Material S1E). Reported values ranged from 0.46 to 9.17 for between-pen transmission [81,82,83,85], 1.05–24.1 for within-pen transmission [81,82,83,85], 1.41–10.8 for between-herd transmission [78,80,90], and 0.8–17.3 for within-herd transmission [23,78,79,86,88,91,93], and 2.92–4.02 for regional- to national-scale transmission [87,94]. Within-pen and within-herd estimates were generally higher than between-pen and between-herd estimates, consistent with greater contact rates in more confined settings. However, substantial variability remained within each transmission scale, and no consistent geographic pattern was observed.

Statistical models were primarily used to identify risk factors (39, 63.9%) [27,60,78,96,97,98,99,100,101,102,103,104,105,106,107,108,109,110,111,112,113,114,115,116,117,118,119,120,121,122,123,124,125,126,127,128,129,130,131] and spatiotemporal patterns (20, 32.8%) [97,116,117,120,127,132,133,134,135,136,137,138,139,140,141,142,143,144,145,146], with less frequent use for outbreak risk estimation [147] or forecasting [148,149,150]. Logistic regression was the dominant approach for identifying risk factors and was used across all locations and host contexts when the response is binary (e.g., infected vs non-infected) [27,60,97,98,99,100,101,102,106,110,113,114,115,116,117,118,121,122,127,128,129,130]. It was notably the near-exclusive method in Africa-based domestic pig studies, where identified risk factors were primarily related to farm management and biosecurity [99,100,101,102,106,113,118]. Tick exposure was examined in three African studies, although it was identified as a significant risk factor only in Uganda [100] and not in studies from Nigeria [99,101]. Bayesian models were most commonly applied in European wild boar studies, particularly hierarchical or spatial models that account for landscape-level uncertainty, spatial autocorrelation, or sparse surveillance data [27,104,105,119]. Risk factors identified through these models tend to be ecological and environmental (e.g., wild boar density, habitat suitability) rather than farm-management variables. When responses were modelled as counts (e.g., outbreaks per area), Poisson or negative binomial regression models were used, with the latter preferred when overdispersion was observed [96,103,108,109,120,126,127,131]. In these studies, identified risk factors tend to be structural or demographic (e.g., number of farms, socioeconomic indices). Kulldorff’s space–time scan statistic [151] was the predominant method for analysing spatiotemporal clustering [97,116,117,120,127,132,133,136,137,138,139,140,141,142,143,144] and has been used consistently since its early application by Iglesias et al. (2016) to detect ASF clusters in wild boar [132]. While it was the sole method applied in domestic pig studies in Asia [117,120,139,140,141,144] and the Dominican Republic [142], kriging and Bayesian space–time models were also used in European wild boar studies [134,135,145,146].

ML models were mainly applied to assess disease suitability (9, 64.3%), often in combination with identifying predictors of suitability. Maximum entropy models (Maxent) were most widely used for suitability estimation, deployed across studies in China [152,153,154], South Korea [155,156], Indonesia [123], Russia [157], and globally [158], commonly using bioclimatic predictors from WorldClim [159]. In Asian studies, population and livestock variables were also found to be important predictors [152,153,154,155]. Tree-based methods (i.e., random forest and classification tree analysis) were applied in two European studies to identify risk factors [104,128]. In China, an ensemble approach combining ten algorithms, including tree-based methods, GLMs, and Maxent, was used to predict suitability using bioclimatic variables, achieving higher accuracy in that study than any individual model considered [160].

To assist in aligning modelling objectives with suitable modelling frameworks, we enumerated the modelling subtypes applied to address each objective (Table 1). For the mechanistic approach, models are described according to model type (compartmental, agent-based, or network model), framework (population-based, individual-based, or metapopulation), uncertainty handling (deterministic, or stochastic), and spatial component (with or without). More detailed information on the specific modelling methods is provided in Supplementary Material S1F.

### 3.3. ASF Data Used in the Papers

Across 151 papers, modelling most commonly focused on domestic pigs (79, 52.3%), followed by wild boar (51, 34%), with the remainder addressing both species (21, 13.9%). Secondary data were the main sources of outbreak information (94, 62.3%), primarily from government veterinary information systems with many of the represented countries maintaining routine national surveillance data. Global sources were also available through the World Animal Health Information System (WAHIS; https://wahis.woah.org/) of the World Organisation for Animal Health (WOAH) and the Global Animal Disease Information System (EMPRES-i; https://empres-i.apps.fao.org/) of the Food and Agriculture Organization (FAO). A smaller number (17, 11.3%) collected primary samples, mainly blood or serum, and mainly detected ASF using real-time PCR or ELISA. The remaining papers (40, 26.5%) based their analyses on mechanistic simulations of hypothetical outbreaks rather than observed outbreak data.

The characteristics of datasets analysed differed between statistical and ML approaches. Among risk factor studies, ML was applied in only three European studies [16,104,128] and one global analysis [167], all of which analysed substantially larger passive surveillance datasets (approximately 2900–16,500 ASF case records) together with remotely sensed environmental, landscape, and climatic predictors. ML was also the predominant approach for environmental suitability studies, which similarly relied on large spatial datasets. In contrast, statistical models were primarily applied to farm-level management and biosecurity studies based on structured epidemiological investigations, including farmer interviews and field surveys. These studies generally included fewer than 1000 observations [100,101,102,110,113,118,121], with several analysing fewer than 100 [116,117,122,129], and commonly employed deliberate epidemiological sampling strategies such as case–control designs.

### 3.4. Explanatory Variables/Input Features Used in Modelling

**Figure 5:**
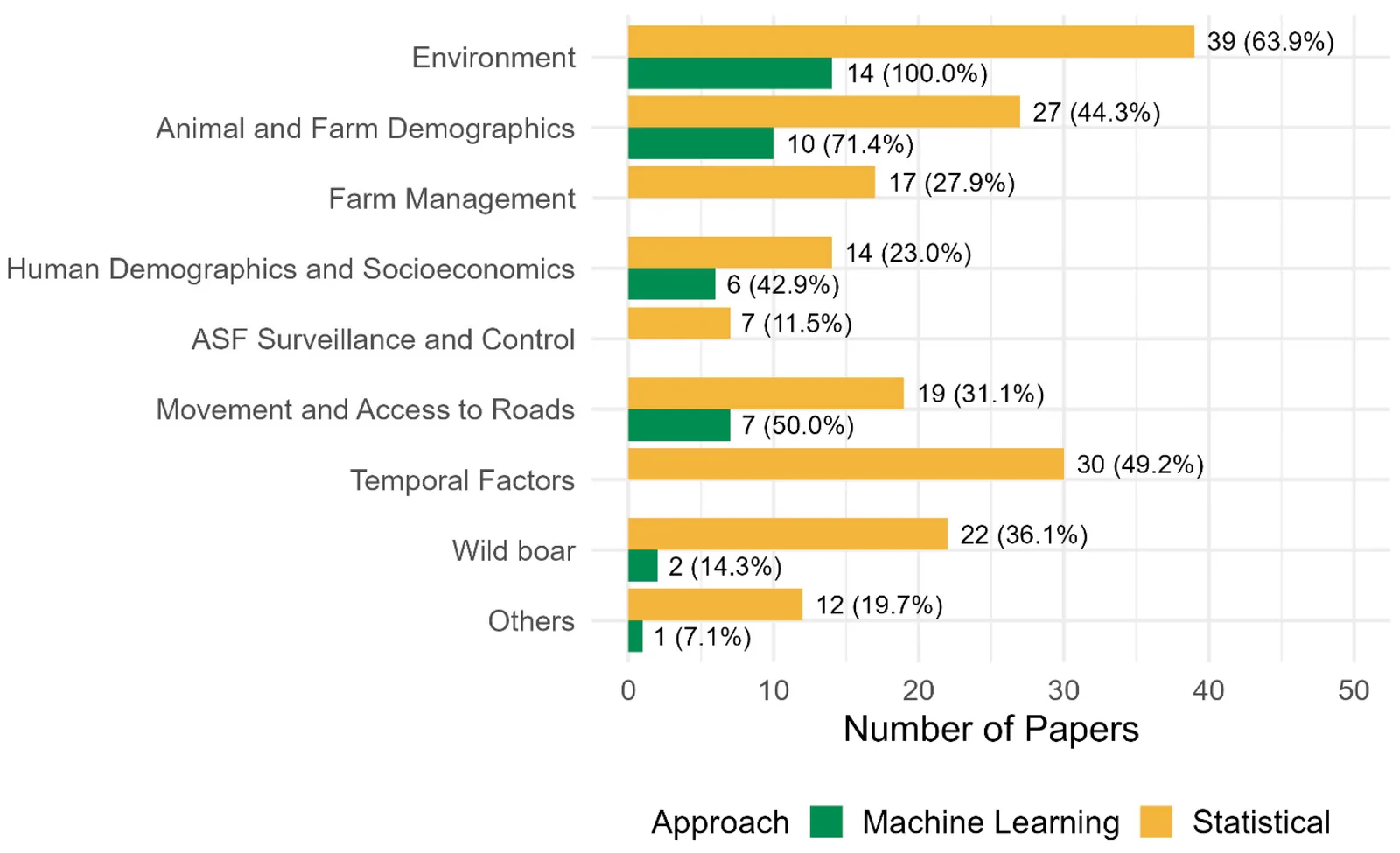
The explanatory variables/input features considered in African swine fever statistical modelling and machine learning papers (1997–2024).

Statistical and ML papers incorporated a broad range of variables for modelling ASF, which are summarised into seven categories (Figure 5). Environmental variables were the most frequently used in both approaches (statistical: 63.9%; ML: 100.0%), including climate (e.g., temperature, precipitation) and landscape features (e.g., elevation, water bodies, forest cover). These were followed by animal and farm demographics (statistical: 44.3%; ML: 71.4%), including pig population, farm density, and animal characteristics (e.g., age, breed). Additional notable variables included human demographics and socioeconomic indicators, movement and road access, and wild boar presence. Use of temporal factors (e.g., month, season), farm management, and ASF surveillance or control variables were reported only in statistical models.

### 3.5. Model Adequacy and Uncertainty Quantification by Approach

Uncertainty quantification and model adequacy reporting were inconsistent across modelling approaches. For mechanistic models, uncertainty quantification was reviewed across three categories: model outputs, estimated parameters, and sensitivity analysis. We found that 12 of 82 papers (14.6%) reported no uncertainty quantification in any of these categories. Uncertainty in model outputs was assessed in 66 papers, excluding 16 that focused solely on parameter estimation. Of these, 31 (47.0%) reported simulation-based uncertainty quantification, all using stochastic models, while four (6.1%) estimated Bayesian credible intervals. The remaining 31 papers (47.0%) did not quantify uncertainty in model outputs. Among the 22 papers that explicitly estimated parameters, 12 (54.5%) used frequentist methods, 10 (45.5%) applied Bayesian methods, and two (9.1%) employed simulation-based approaches. Sensitivity analysis was conducted in 43 of 82 papers (52.4%), mainly to examine the influence of model parameters (38, 46.3%), while the remainder assessed model assumptions and initial conditions.

For statistical models, model adequacy assessment, assumption checking, and model selection procedures were reviewed across all 61 papers. Model adequacy was not reported in 30 papers (49.2%). Among those that reported an assessment, the most common metrics were the Akaike Information Criterion (AIC; 13, 21.3%), primarily for model selection, and the area under the receiver operating characteristic curve (AUC-ROC; 8, 13.1%) for evaluating the discriminative ability of binary prediction models. Model assumptions were not assessed in 31 papers (50.8%). Among those that reported assumption checking, the most common assessments were multicollinearity (13, 21.3%), typically using variance inflation factors (VIFs), spatial autocorrelation (10, 16.4%), commonly using Moran’s I or semivariograms, and normality of residuals (5, 8.2%) using formal or graphical methods. Model selection procedures were evaluated in 47 papers (77.0%), excluding studies for which model selection was not applicable, such as space–time clustering analyses. Of these, 23 (48.9%) used stepwise selection, including backward (11, 23.4%), forward (6, 12.8%), and bidirectional (6, 12.8%) approaches. Fifteen papers (31.9%) retained all candidate variables in the final model, while six (12.8%) selected variables based on univariable screening using a predefined significance threshold.

For the 14 ML papers, we reviewed the same three dimensions as for statistical models, and additionally examined feature importance or interpretability, data splitting, and uncertainty quantification. Model adequacy was reported in all papers, with AUC-ROC (12, 85.7%) and the True Skill Statistic (5, 35.7%) being the most common metrics. Model assumptions were assessed in nine papers (64.3%), most often multicollinearity using VIFs, particularly in MaxEnt-based suitability analyses. Model selection primarily involved retaining variables that met underlying assumptions (6, 42.9%) or exceeded a minimum contribution threshold (5, 35.7%). All papers reported feature importance or interpretability, most commonly using jackknife tests (6, 42.9%), predominantly in MaxEnt models, and relative contribution metrics (4, 28.6%), mainly in ensemble models. Most papers (11, 78.6%) used a two-way data split, typically 75:25 or 80:20, for training and validation/testing, while the remainder applied cross-validation or bootstrapping. Uncertainty quantification was infrequent, with nine papers (64.3%) not reporting it. Among those that did, simulation/sampling-based methods were most commonly applied (3, 21.4%).

A full list of information extracted is available in Supplementary Material S1G.

### 3.6. Clustering Analysis

HCPC identified three clusters that largely corresponded to the major modelling approaches. The analysis further characterised each approach by its most commonly associated objectives and study locations (Table 2). Cluster 1 was characterised by mechanistic papers that primarily assessed control strategies and transmission determinants in simulated or non-specific settings. Cluster 2 was dominated by statistical papers focused on identifying risk factors and spatiotemporal patterns in Europe and Africa. Cluster 3 comprised ML papers that estimated suitability for disease occurrence in Asia and globally. Mean publication year was similar across clusters (Cluster 1: 2021; Cluster 2: 2020; Cluster 3: 2021), with no significant difference in publication year among clusters (Kruskal–Wallis, *p* = 0.5022). Full HCPC category enrichment and depletion results are provided in Supplementary Material S1H.

**Figure 6:**
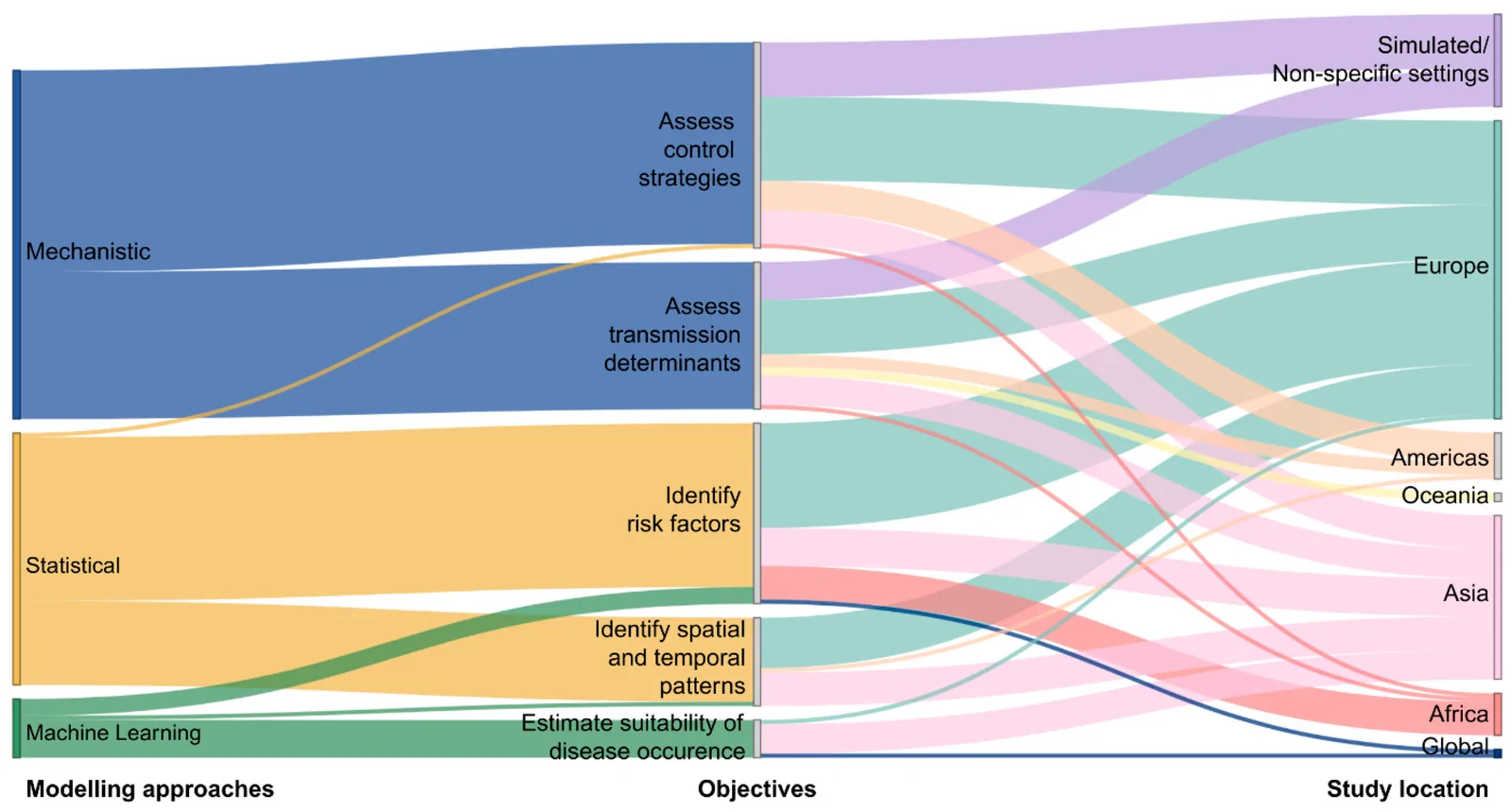
Sankey diagram showing the distribution of African swine fever (ASF) modelling papers by modelling approach, study objective, and study location. The weight of each link is proportional to the number of papers. Links between modelling approaches (**left**) and objectives (**middle**) are coloured based on approach, while links between modelling objectives (**middle**) and study location (**right**) are coloured based on study location.

While these clusters represent the most characteristic combinations of study attributes, they are not mutually exclusive. For example, modelling approaches are well represented across multiple geographic regions despite the strong associations between clusters and particular study locations (Figure 6). Mechanistic models were widely used in European and Asian settings despite their strong association with non-specific settings, while statistical studies, associated with Europe and Africa, were also frequently conducted in Asia.

## 4. Discussion

Within the included literature, interest in ASF modelling increased from 2011 onwards, particularly across Europe, Asia, and Africa, with mechanistic and statistical approaches dominating throughout the study period and machine learning gaining traction in recent years. Modelling activity increased substantially from 2020 onwards as ASF expanded across Asia and Oceania and re-emerged in the Americas and Europe [169]. Despite this rapid growth, this review found that mechanistic, statistical, and ML approaches have continued to serve complementary roles across different epidemiological settings, with regional variation reflected in modelling priorities. Important gaps nevertheless remain, including the underrepresentation of affected regions, limited representation of some transmission pathways, and inconsistent reporting of model evaluation practices.

Differences in ecology and production systems were reflected in the transmission pathways represented across regions. Studies from sub-Saharan Africa and Asia often focused on biosecurity practices and trade networks [30,65,71,99,100,101,102,106,113,118], likely reflecting the predominance of smallholder production systems and transmission pathways that are less relevant in intensively managed commercial systems. In contrast, European studies placed greater emphasis on wildlife reservoirs and transmission at the wildlife–livestock interface [26,27,43,61,62,64,68,72,75,76,77,104,105,119]. These findings highlight the importance of context-specific investigations, as regional differences in ecology, production systems, and socioeconomic conditions shape ASF transmission and may limit the transferability of findings across settings.

The substantial heterogeneity in reported $R_{0}$ estimates further highlights the context dependence of ASF transmission. Estimates varied not only across geographic settings but also according to the transmission scale examined, including within-pen, between-pen, within-herd, between-herd, and regional- to national-scale transmission [23,78,79,80,81,82,83,85,86,87,88,90,91,93,94]. These differences complicate direct comparisons of transmissibility across studies and underscore the importance of interpreting $R_{0}$ estimates within their epidemiological context rather than as universally comparable measures of transmission potential. Consequently, control thresholds and intervention strategies should be informed by estimates derived from transmission settings that are relevant to the production system and epidemiological context under investigation.

Although ASF modelling activity increased substantially following the global spread of the disease, HCPC identified no temporal separation of studies, indicating that the relationships between research objectives, modelling approaches, and geographic settings remained relatively consistent over time. This finding suggests that the growth of the literature has been driven by the continued investigation of a set of recurring epidemiological questions across diverse outbreak settings, rather than by fundamental shifts in methodological preference. Mechanistic, statistical, and ML approaches have therefore developed complementary roles, with methodological choice largely determined by the analytical requirements of the research question and the available data. Within the ASF modelling literature, this is reflected in the predominant application of mechanistic models to transmission dynamics and intervention studies, statistical models to epidemiological risk factor analyses, and ML to environmental suitability modelling. Nevertheless, while a small number of ASF papers applied more than one modelling approach within a single investigation, these approaches were generally used to address distinct research questions rather than integrated to address a common modelling objective.

In the broader modelling literature, the integration of modelling approaches to leverage their respective strengths has been explored through a range of methodological frameworks, with new approaches continuing to emerge. Established examples include coupling mechanistic models with Bayesian statistical inference for parameter estimation and uncertainty quantification [170], as also performed in several of the ASF papers included in this review. Similarly, ensemble methods combine predictions from multiple modelling approaches to improve predictive performance, as demonstrated by Li et al. (2022) [160], who integrated statistical regression and ML models to predict ASF spread. Beyond combining complementary methods within a broader modelling framework, recent developments increasingly seek to embed these approaches within a single modelling architecture. For example, the emerging field of scientific machine learning (SciML) integrates mechanistic models with ML algorithms to improve predictive performance while preserving biological realism [171]. Although such approaches remain largely unexplored within the ASF modelling literature identified in this review, they illustrate the continuing evolution of methodologies towards closer integration of modelling approaches, providing further opportunities for methodological innovation in ASF research.

The expansion of mechanistic modelling since the review by Hayes et al. [9] reflects a broadening of ASF models towards more comprehensive representation of the ecological and epidemiological processes underlying disease spread. This is evident in the incorporation of multiple host systems, diverse transmission pathways, and spatially explicit population structures, enabling more integrated representation of ASF transmission across varied epidemiological settings. Despite these developments, inconsistent reporting of uncertainty quantification of model outputs and estimated parameters continues to limit assessment of model robustness.

The contrasting use of statistical and ML approaches was accompanied by differences in the characteristics of the underlying datasets. Statistical models remain well suited to structured epidemiological investigations, where stronger structural assumptions and fewer parameters allow robust and interpretable inference from relatively small but carefully sampled datasets. However, many statistical studies did not report model diagnostics or assumption checks, raising concerns about the reliability of reported associations given that valid statistical inference depends on appropriate model specification and evaluation. In contrast, ML applications in ASF have largely been confined to environmental suitability analyses based on large passive surveillance and spatial datasets. This limited scope is consistent with the broader synthesis by Keshavamurthy et al. (2022), who reported comparatively limited use of ML in non-zoonotic livestock diseases such as ASF despite the wider application of ML to temporal forecasting and disease risk prediction in other infectious disease systems [8]. Collectively, these findings demonstrate that statistical and ML approaches have occupied complementary roles within ASF research, with each predominating in distinct data contexts.

Despite the importance of regional ecological context, one notable gap identified in this review is the limited representation of tick-mediated transmission in endemic African settings. Although ticks have been incorporated into several mechanistic models, these applications were confined to hypothetical outbreak scenarios rather than studies representing the sylvatic transmission cycle in Africa. In contrast, African mechanistic models acknowledged ticks in the epidemiological context but did not explicitly represent them within the model structure, while statistical studies provided limited and inconsistent evidence for their role as a risk factor. Although these findings do not establish the epidemiological importance of ticks across African production systems, they suggest that tick-mediated transmission remains underrepresented in modelling studies from regions where the sylvatic cycle is recognised to contribute to ASF persistence.

Another important gap identified in this review is the geographic underrepresentation of ASF modelling studies, particularly in affected countries across Asia and Oceania. Given the substantial regional differences in production systems, ecology, and transmission pathways observed across the literature, this limits the availability of locally relevant evidence to inform preparedness and control strategies in these settings. Although the reasons for this underrepresentation were not examined in the present review, previous assessments suggest that limited surveillance systems, veterinary infrastructure, and research capacity may contribute to the scarcity of modelling studies in some affected countries [172,173,174,175]. These constraints can reduce the availability of high-quality epidemiological data while limiting the technical and institutional capacity required to develop and apply context-specific models. However, the extent of this underrepresentation may have been influenced by the use of English-language bibliographic databases, which may not have captured relevant studies indexed exclusively in regional or non-English databases.

While this review provides a comprehensive overview of ASF modelling, several limitations should be considered when interpreting its findings. First, we restricted inclusion to papers written in English and published as journal articles, and excluded preprints, conference papers, books, dissertations, and theses, which may have resulted in the omission of important, relevant work. Second, we excluded studies that used statistical techniques but did not meet our criteria for quantitative modelling. While this improved comparability between approaches, it also means the papers included here may not fully represent the breadth of statistical applications to ASF. Third, while some traditional regression-based analyses included in this review could also be classified under broader definitions of ML, we distinguished them to clarify contrasts between conventional statistical approaches and algorithmic, data-driven ML methods. Fourth, the summary of reported *R*_0_ estimates is descriptive and limited to the mechanistic studies included in this scoping review and does not represent a comprehensive systematic synthesis of all published ASF *R*_0_ estimates. Taken together, these limitations indicate that our findings should be interpreted as a structured overview rather than an exhaustive account of all quantitative analyses conducted on ASF.

## 5. Conclusions

The ASF modelling literature is characterised by complementary modelling paradigms, with different approaches addressing distinct epidemiological questions under different data and epidemiological contexts. Despite considerable advances in the scope and complexity of ASF modelling, important gaps remain, including the underrepresentation of affected regions, limited representation of ticks as a transmission pathway, and inconsistent reporting of model evaluation practices. By integrating these findings into a practical framework linking modelling objectives, methodological approaches, and epidemiological contexts, this review provides a valuable reference for the design of future ASF modelling studies.

## 6. Future Directions

This review highlights several opportunities to strengthen future ASF modelling research. The complementary roles identified in this review suggest potential value in integrating modelling approaches, drawing on the strengths of each to address complex epidemiological questions that may not be adequately captured by a single framework. Emerging methodological developments that integrate complementary modelling approaches within a single modelling architecture may provide additional opportunities for future ASF modelling research. As ASF continues to spread into new settings, greater emphasis on context-specific modelling will be important to capture regional differences in production systems, wildlife ecology, and transmission pathways. Expanding modelling efforts in currently underrepresented regions and better representing transmission pathways that remain infrequently modelled would improve understanding of the diverse epidemiological contexts in which ASF persists and spreads. Beyond methodological development, dedicated systematic reviews synthesising key epidemiological parameters, such as reported basic reproduction number (*R*_0_) estimates, would improve understanding of how transmission varies across production systems, host populations, and epidemiological settings. Across all modelling approaches, more consistent reporting of model evaluation, including appropriate model adequacy assessment, assumption checking, and uncertainty quantification, would improve the transparency, robustness, and comparability of future studies. Collectively, this review provides a practical reference for designing future ASF modelling studies and an evidence base for selecting modelling strategies aligned with specific research objectives.

## Figures and Tables

**Table 1:** Methods applied to different African swine fever (ASF) modelling objectives and the total number of papers addressing that objective. For mechanistic models, each row represents a distinct combination of model type (CM/ABM/NM), modelling framework (PB/IB/Met), approach to uncertainty (deterministic/stochastic), and spatial representation (spatial/non-spatial). Blue cells indicate characteristics present in that combination, while blank cells indicate characteristics that were not used. CM—compartmental model; ABM—agent-based model; NM—network model; PB—population-based; IB—individual-based; Metapop—metapopulation framework.

Objective (Number of Papers, *n*)	Approach/Model Characteristics										Count	References
	**Mechanistic modelling**											
	Model Type			Modelling framework			Approach to uncertainty		Spatial representation			
	CM	ABM	NM	PB	IB	Metapop	Deterministic	Stochastic	Spatial	Non-Spatial		
Assess control strategies (*n* = 40)											19	[17,18,20,22,27,29,34,35,38,40,41,42,45,46,50,52,53,54,56]
											14	[17,19,23,24,28,30,31,33,40,42,43,44,54,55]
											7	[21,25,32,37,48,49,51]
	Others										5	[20,26,36,39,47]
Assess transmission determinants (*n* = 34)											14	[19,24,28,30,33,43,63,66,69,70,71,73,74,77]
											10	[29,34,46,53,58,60,65,67,72,77]
											9	[32,51,57,61,62,64,68,75,76]
	Others										2	[26,58]
Estimate parameters (*n* = 22)											12	[23,44,71,73,78,79,80,87,90,91,93,94]
											4	[29,84,85,89]
											4	[81,82,83,86]
											2	[88,92]
Assess consequences of outbreaks (*n* = 9)											6	[40,41,42,54,58,77]
											5	[40,42,54,77,95]
											2	[25,57]
Assess model assumptions (*n* = 5)											2	[17,161]
											2	[85,162]
	Others										2	[17,163]
Forecast spread (*n* = 4)											3	[20,35,52]
											2	[35,48]
	**Statistical modelling**											
Identify risk factors (*n* = 39)	Logistic regression										22	[27,60,97,98,99,100,101,102,106,110,113,114,115,116,117,118,121,122,127,128,129,130]
	Linear regression										5	[78,98,123,124,125]
	Bayesian model										4	[27,104,111,119]
	Other GLM extensions										4	[105,107,112,131]
	Poisson regression										4	[96,103,126,131]
	Negative binomial regression										4	[108,109,120,127]
	Survival model										1	[111]
Identify spatial and temporal patterns (*n* = 20)	Space–time clustering										16	[97,116,117,120,127,132,133,136,137,138,139,140,141,142,143,144]
	Bayesian model										2	[134,146]
	Spatial model										2	[135,145]
Forecast spread (*n* = 3)	Logistic model										1	[148]
	Other GLM extensions										1	[149]
	Poisson regression										1	[150]
Identify temporal trends (*n* = 2)	Bayesian model										1	[164]
	Linear regression										1	[98]
Assess control strategies (*n* = 1)	Bayesian model										1	[165]
Assess transmission determinants (*n* = 1)	Other GLM extensions										1	[166]
Estimate outbreak risk (*n* = 1)	Risk assessment model										1	[147]
	**Machine learning**											
Estimate suitability of disease occurrence (*n* = 9)	Maximum entropy modelling										8	[123,152,153,154,155,156,157,158]
	Ensemble modelling										1	[160]
Identify risk factors (*n* = 4)	Tree-based approach										4	[16,104,128,167]
Identify spatial and temporal patterns (*n* = 1)	Space–time clustering (ML-based)										1	[16]
Forecast spread (*n* = 1)	Multiple models										1	[168]

**Table 2:** Selected Hierarchical Clustering on Principle Components (HCPC) categories predominantly characterising each cluster of African swine fever (ASF) modelling papers, based on category enrichment analysis.

Cluster	Category	Level	Mod/Cla (%)	Cla/Mod (%)	Global (%)	*v*-Test	*p*-Value
1	Approach	Mechanistic	100	100	53	16.9	<0.001
1	Objective	Assess control strategies	97.6	35.1	19.1	6.9	<0.001
1	Objective	Assess transmission determinants	100	29.8	15.8	6.7	<0.001
1	Continent	Simulated/Non-specific settings	100	26.3	14	6.2	<0.001
2	Approach	Statistical	100	95.1	36.3	15.5	<0.001
2	Objective	Identify risk factors	98	58.5	22.8	10.2	<0.001
2	Objective	Identify spatial and temporal patterns	100	26.8	10.2	6.5	<0.001
2	Continent	Europe	54.3	61	42.8	4.2	<0.001
2	Continent	Africa	72.7	9.8	5.1	2.3	0.022
3	Approach	Machine learning	82.6	100	10.7	10	<0.001
3	Objective	Estimate suitability of disease occurrence	100	47.4	4.2	6.6	<0.001
3	Continent	Asia	23	73.7	28.4	4.2	<0.001
3	Continent	Global	100	21.1	1.9	4.1	<0.001

Mod/Cla (%), Modality/Class: percentage of papers with a given category that belong to the cluster; Cla/Mod (%), Class/Modality: percentage of papers within the cluster that have the given category; Global (%), percentage of all papers with the given category.

## Data Availability

The data supporting the findings of this study are available in Supplementary Material S2 (.xlsx), which contains the complete list of included studies and the extracted data used in the analyses. No additional datasets were generated during the study.

## Supplementary Materials

The following supporting information can be downloaded at: https://www.mdpi.com/article/10.3390/tropicalmed11080228/s1, Supplementary Material S1 (PDF); S1A: PRISMA-ScR checklist; S1B: Full search strategy; S1C: Continent-level geographic distribution of papers; S1D: Temporal distribution of papers by model subtypes; S1E: Estimated basic reproductive number; S1F: Detailed list of methods applied per objective; S1G: Model adequacy and uncertainty quantification by approach; S1H: Clustering analysis using HCPC; Supplementary Material S2 (.xlsx)—Raw dataset (Complete list of papers and extracted information) [13,16,17,18,19,20,21,22,23,24,25,26,27,28,29,30,31,32,33,34,35,36,37,38,39,40,41,42,43,44,45,46,47,48,49,50,51,52,53,54,55,56,58,59,60,61,62,63,64,65,66,67,68,69,70,71,72,73,74,75,76,77,78,79,80,81,82,83,84,85,86,87,88,89,90,91,92,93,94,95,96,97,98,99,100,101,102,103,104,105,106,107,108,109,110,111,112,113,114,115,116,117,118,119,120,121,122,123,125,126,127,128,129,130,131,132,133,134,135,136,137,138,139,140,141,142,143,144,145,146,147,148,149,150,152,153,154,155,156,157,158,160,161,162,163,164,165,166,167,168].
