# Supplementary material for: Modelling African Swine Fever Transmission and Epidemiology: A Scoping Review of Mechanistic, Statistical, and Machine Learning Approaches
Source: Trop Med Infect Dis. 2026 Aug 14;11(8):228. doi: 10.3390/tropicalmed11080228 (PMC13517675; doi:10.3390/tropicalmed11080228)
Supplement: Supplementary file 1 [file tropicalmed-11-00228-s001.zip › Supp Mat S1 - ASF_Modelling_Scoping_Review - tropicalmed.pdf]

SUPPLEMENTARY MATERIAL S1

Modelling African Swine Fever Transmission and Epidemiology: A Scoping Review of Mechanistic, Statistical, and Machine Learning Approaches

Kim Dianne B. Ligue-Sabio[1,2]*, Yoni Nazarathy[3], Kien Quoc Do[4], Luis Furuya-Kanamori[1], Yusuf A. Sucol[5], Benn Sartorius[1], Colleen L. Lau[1]

[1] Frazer Institute, Faculty of Health, Medicine and Behavioural Sciences, The University of Queensland, Brisbane, QLD 4029, Australia
[2]Department of Mathematics, Physics, and Computer Science, College of Science and Mathematics, University of the Philippines Mindanao, Davao City 8022, Philippines
[3]School of Mathematics and Physics, Faculty of Science, The University of Queensland, Brisbane, QLD 4072, Australia
[4]School of Public Health, Faculty of Health, Medicine and Behavioural Sciences, The University of Queensland, Brisbane, QLD 4006, Australia
[5]UPLB Climate and Disaster Risks Studies Center, School of Environmental Science and Management, University of the Philippines Los Baños, Los Baños 4031, Philippines

*Correspondence: k.ligue@uq.edu.au

Section headings in this document are **BOOKMARKED** in the PDF to assist navigation.

S1A. PRISMA-ScR checklist

Supplementary Table S1. PRISMA-ScR checklist Preferred Reporting Items for Systematic reviews and Meta-Analyses extension for Scoping Reviews (PRISMA-ScR) Checklist

SECTION	ITEM	PRISMA-ScR CHECKLIST ITEM	REPORTED ON PAGE #
		**TITLE**	
Title	1	Identify the report as a scoping review.	1
		**ABSTRACT**	
Structured summary	2	Provide a structured summary that includes (as applicable): background, objectives, eligibility criteria, sources of evidence, charting methods, results, and conclusions that relate to the review questions and objectives.	1
		**INTRODUCTION**	
Rationale	3	Describe the rationale for the review in the context of what is already known. Explain why the review questions/objectives lend themselves to a scoping review approach.	3
Objectives	4	Provide an explicit statement of the questions and objectives being addressed with reference to their key elements (e.g., population or participants, concepts, and context) or other relevant key elements used to conceptualize the review questions and/or objectives.	3
		**METHODS**	
Protocol and registration	5	Indicate whether a review protocol exists; state if and where it can be accessed (e.g., a Web address); and if available, provide registration information, including the registration number.	3
Eligibility criteria	6	Specify characteristics of the sources of evidence used as eligibility criteria (e.g., years considered, language, and publication status), and provide a rationale.	3-4
Information sources*	7	Describe all information sources in the search (e.g., databases with dates of coverage and contact with authors to identify additional sources), as well as the date the most recent search was executed.	4
Search	8	Present the full electronic search strategy for at least 1 database, including any limits used, such that it could be repeated.	4; Supplementary material 1B
Selection of sources of evidence†	9	State the process for selecting sources of evidence (i.e., screening and eligibility) included in the scoping review.	4
Data charting process‡	10	Describe the methods of charting data from the included sources of evidence (e.g., calibrated forms or forms that have been tested by the team before their use, and whether data charting was done independently or in duplicate) and any processes for obtaining and confirming data from investigators.	4
Data items	11	List and define all variables for which data were sought and any assumptions and simplifications made.	4
Critical appraisal of individual	12	If done, provide a rationale for conducting a critical appraisal of included sources of evidence; describe the methods used and how	NA

SECTION	ITEM	PRISMA-ScR CHECKLIST ITEM	REPORTED ON PAGE #
sources of evidence§		this information was used in any data synthesis (if appropriate).	
Synthesis of results	13	Describe the methods of handling and summarizing the data that were charted.	4-5
		**RESULTS**	
Selection of sources of evidence	14	Give numbers of sources of evidence screened, assessed for eligibility, and included in the review, with reasons for exclusions at each stage, ideally using a flow diagram.	5
Characteristics of sources of evidence	15	For each source of evidence, present characteristics for which data were charted and provide the citations.	Supplementary material 2
Critical appraisal within sources of evidence	16	If done, present data on critical appraisal of included sources of evidence (see item 12).	NA
Results of individual sources of evidence	17	For each included source of evidence, present the relevant data that were charted that relate to the review questions and objectives.	Supplementary material 2
Synthesis of results	18	Summarize and/or present the charting results as they relate to the review questions and objectives.	5-15
		**DISCUSSION**	
Summary of evidence	19	Summarize the main results (including an overview of concepts, themes, and types of evidence available), link to the review questions and objectives, and consider the relevance to key groups.	15-18
Limitations	20	Discuss the limitations of the scoping review process.	18
Conclusions	21	Provide a general interpretation of the results with respect to the review questions and objectives, as well as potential implications and/or next steps.	18
		**FUNDING**	
Funding	22	Describe sources of funding for the included sources of evidence, as well as sources of funding for the scoping review. Describe the role of the funders of the scoping review.	19

JBI = Joanna Briggs Institute; PRISMA-ScR = Preferred Reporting Items for Systematic reviews and Meta-Analyses extension for Scoping Reviews.

* Where *sources of evidence* (see second footnote) are compiled from, such as bibliographic databases, social media platforms, and Web sites.

† A more inclusive/heterogeneous term used to account for the different types of evidence or data sources (e.g., quantitative and/or qualitative research, expert opinion, and policy documents) that may be eligible in a scoping review as opposed to only studies. This is not to be confused with *information sources* (see first footnote).

‡ The frameworks by Arksey and O'Malley (6) and Levac and colleagues (7) and the JBI guidance (4, 5) refer to the process of data extraction in a scoping review as data charting.

§ The process of systematically examining research evidence to assess its validity, results, and relevance before using it to inform a decision. This term is used for items 12 and 19 instead of "risk of bias" (which is more applicable to systematic reviews of interventions) to include and acknowledge the various sources of evidence that may be used in a scoping review (e.g., quantitative and/or qualitative research, expert opinion, and policy document). [13]

S1B. Full search strategy

S1B.1. PICO framework

The PICO framework was applied to identify core concepts and keywords for developing the search strategy:

**P – Population**<br>African swine fever<br><br>**I – Intervention**<br>Mathematical model<br>Epidemiological models<br>Mechanistic models<br>Deterministic models<br>Stochastic models<br>Compartmental models<br>Agent-based models<br>Simulation<br><br>Statistical models<br>Regression analysis<br>Linear models<br>Logistic models<br>Bayesian estimation<br>Likelihood functions<br>Spatial models<br>Multivariate/Multivariable models<br>Mixed models<br>Random Effects<br>Survival analysis<br>Risk factor analysis<br>Hierarchical models<br>Statistical significance	Machine learning<br>Artificial Intelligence<br>Supervised machine learning<br>Unsupervised machine learning<br>Deep learning<br>Neural network<br>Ensemble<br><br>**C – Comparison**<br>N/A<br><br>**O – Outcome**<br>Not limited during search<br><br>**Exclusion Criteria:** Towards the development of vaccine<br><br>Genotype<br>Genomics<br>Proteins<br>Proteomics<br>Transcriptome<br>Transcriptomics<br>Lipids<br>Lipidomics

Using these keywords, the full search strategy was tailored to the syntax requirements of each database.

S1B.2. PubMed

("African Swine Fever" [TIAB] OR "African Swine Fever"[Mesh])

AND

(mathematical [TIAB] OR mechanistic [TIAB] OR epidemiolog* [TIAB] OR deterministic [TIAB] OR stochastic [TIAB] OR compartmental [TIAB] OR agent-based [TIAB] OR agent [TIAB] OR simulat* [TIAB] OR statistic* [TIAB] OR regression* [TIAB] OR linear [TIAB] OR logistic [TIAB] OR baye* OR likelihood [TIAB] OR spatial [TIAB] OR multivar* [TIAB] OR mixed [TIAB] OR "random effects" [TIAB] OR survival [TIAB] OR risk* [TIAB] OR hierarchical [TIAB] OR significan* OR "machine learning" [TIAB] OR "artificial intelligence" [TIAB] OR AI [TIAB] OR "supervi*ed" [TIAB] OR "unsupervi*ed" [TIAB] OR "deep learning" [TIAB] OR "neutral network*" [TIAB] OR ensemble [TIAB] OR model* [TIAB] OR

"Epidemiological Models"[Mesh] OR
“Compartmental Models”[Mesh] OR
"Statistics as Topic"[Mesh] OR
"Forecasting"[Mesh] OR

"Machine Learning"[Mesh] OR
“Computer Simulation”[Mesh])

NOT
(genom* [TIAB] OR genotyp* [TIAB] OR transcriptom* [TIAB] OR protein* [TIAB] OR proteomic* [TIAB] OR lipid* [TIAB])

S1B.3. Embase

('African Swine Fever':ti,ab OR 'African Swine Fever'/exp)

AND

(mathematical:ti,ab OR mechanistic:ti,ab OR epidemiolog*:ti,ab OR deterministic:ti,ab OR stochastic:ti,ab OR compartmental:ti,ab OR agent-based:ti,ab OR agent:ti,ab OR simulat*:ti,ab OR statistic*:ti,ab OR regression*:ti,ab OR linear:ti,ab OR logistic:ti,ab OR baye* OR likelihood:ti,ab OR spatial:ti,ab OR multivar*:ti,ab OR mixed:ti,ab OR 'random effects':ti,ab OR survival:ti,ab OR risk*:ti,ab OR hierarchical:ti,ab OR significan* OR 'machine learning':ti,ab OR 'artificial intelligence':ti,ab OR AI:ti,ab OR supervi$ed:ti,ab OR unsupervi$ed:ti,ab OR 'deep learning':ti,ab OR 'neutral network*':ti,ab OR ensemble:ti,ab OR model*:ti,ab OR

'mathematical model'/exp OR
'epidemiological model'/exp OR
'mechanistic model'/exp OR
'compartmental model'/exp OR
'statistical analysis'/exp OR
'machine learning'/exp OR
'simulation'/exp)

NOT
(genom*:ti,ab OR genotyp*:ti,ab OR transcriptom*:ti,ab OR protein*:ti,ab OR proteomic*:ti,ab OR lipid*:ti,ab)

S1B.4. Web of Science

("African Swine Fever")
AND
(mathematical OR mechanistic OR epidemiolog* OR deterministic OR stochastic OR compartmental OR agent-based OR agent OR simulat* OR statistic* OR regression* OR linear OR logistic OR baye* OR likelihood OR spatial OR multivar* OR mixed OR "random effects" OR survival OR risk* OR hierarchical OR significan* OR "machine learning" OR "artificial intelligence" OR AI OR supervi$ed OR unsupervi$ed OR "deep learning" OR "neutral network*" OR ensemble OR model*)
NOT
(genom* OR genotyp* OR transcriptom* OR protein* OR proteomic* OR lipid*)

S1B.5. Scopus

(TITLE-ABS("African Swine Fever") OR INDEXTERMS("African Swine Fever"))
AND
(TITLE-ABS(mathematical) OR TITLE-ABS(epidemiolog*) OR TITLE-ABS(deterministic) OR TITLE-ABS(stochastic) OR TITLE-ABS(compartmental) OR TITLE-ABS(agent-based) OR TITLE-ABS(agent) OR TITLE-ABS(simulat*) OR TITLE-ABS(statistic*) OR TITLE-ABS(regression*) OR TITLE-

ABS(linear) OR TITLE-ABS(logistic) OR TITLE-ABS(baye*) OR TITLE-ABS(likelihood) OR TITLE-ABS(spatial) OR TITLE-ABS(multivar*) OR TITLE-ABS(mixed) OR TITLE-ABS("random effects") OR TITLE-ABS(survival) OR TITLE-ABS(risk*) OR TITLE-ABS(hierarchical) OR TITLE-ABS(significan*) OR TITLE-ABS("machine learning") OR TITLE-ABS("artificial intelligence") OR TITLE-ABS(AI) OR TITLE-ABS(supervi*ed) OR TITLE-ABS(unsupervi*ed) OR TITLE-ABS("deep learning") OR TITLE-ABS("neutral network*") OR TITLE-ABS(ensemble) OR TITLE-ABS(model*) OR

INDEXTERMS("mathematical model") OR
INDEXTERMS("epidemiological model") OR
INDEXTERMS("mechanistic model") OR
INDEXTERMS("compartmental model") OR
INDEXTERMS("statistical analysis") OR
INDEXTERMS("machine learning") OR
INDEXTERMS(simulation))

AND NOT
(TITLE-ABS(genom*) OR TITLE-ABS(genotyp*) OR TITLE-ABS(transcriptom*) OR TITLE-ABS(protein*) OR TITLE-ABS(proteomic*) OR TITLE-ABS(lipid*))

S1C. Continent-level geographic distribution of papers

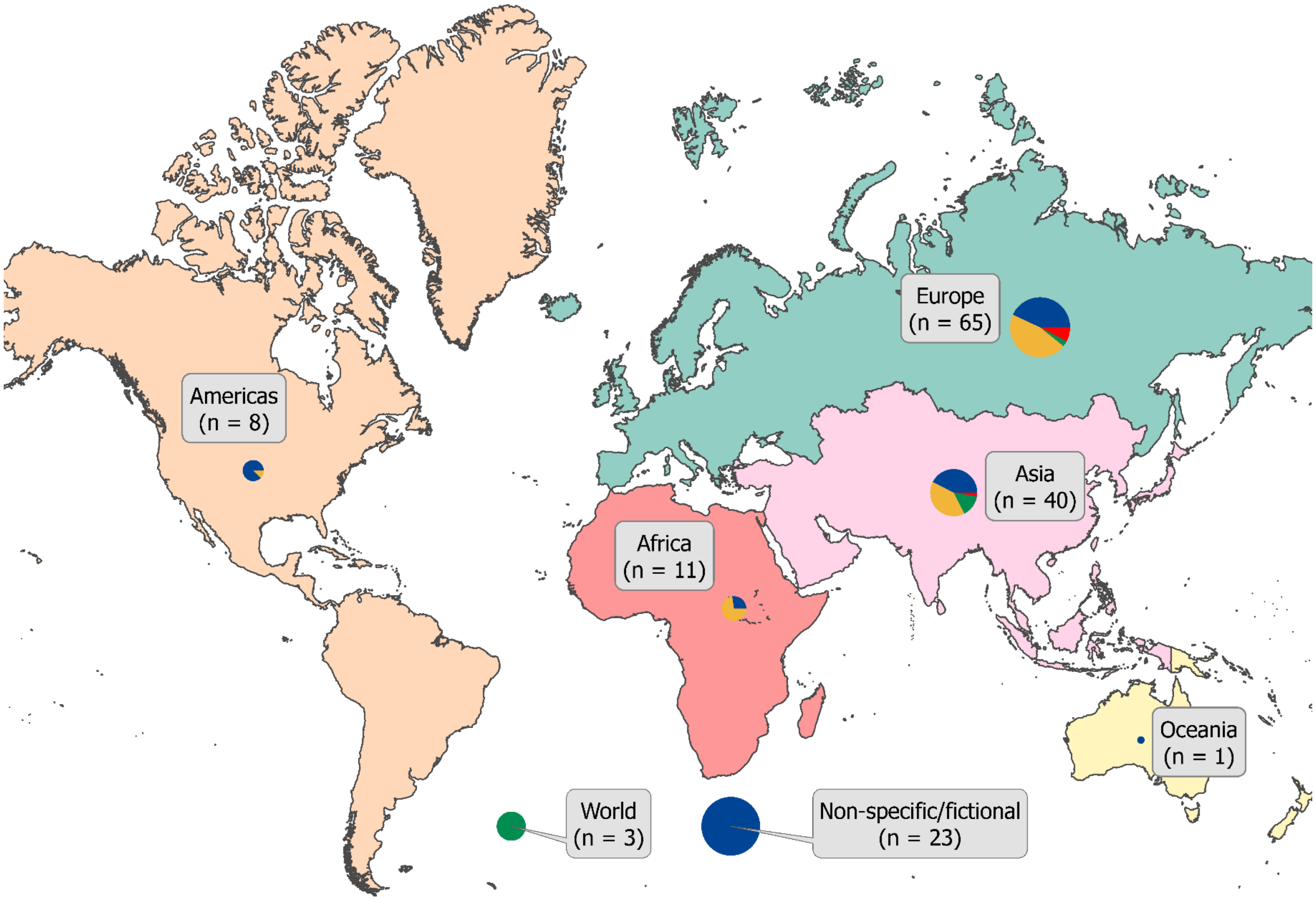

Supplementary Figure S1. Distribution of ASF modelling studies by geographic focus at the continent level. Pie chart symbol size indicates relative counts by approach per continent.

S1D. Temporal distribution of papers by model subtypes

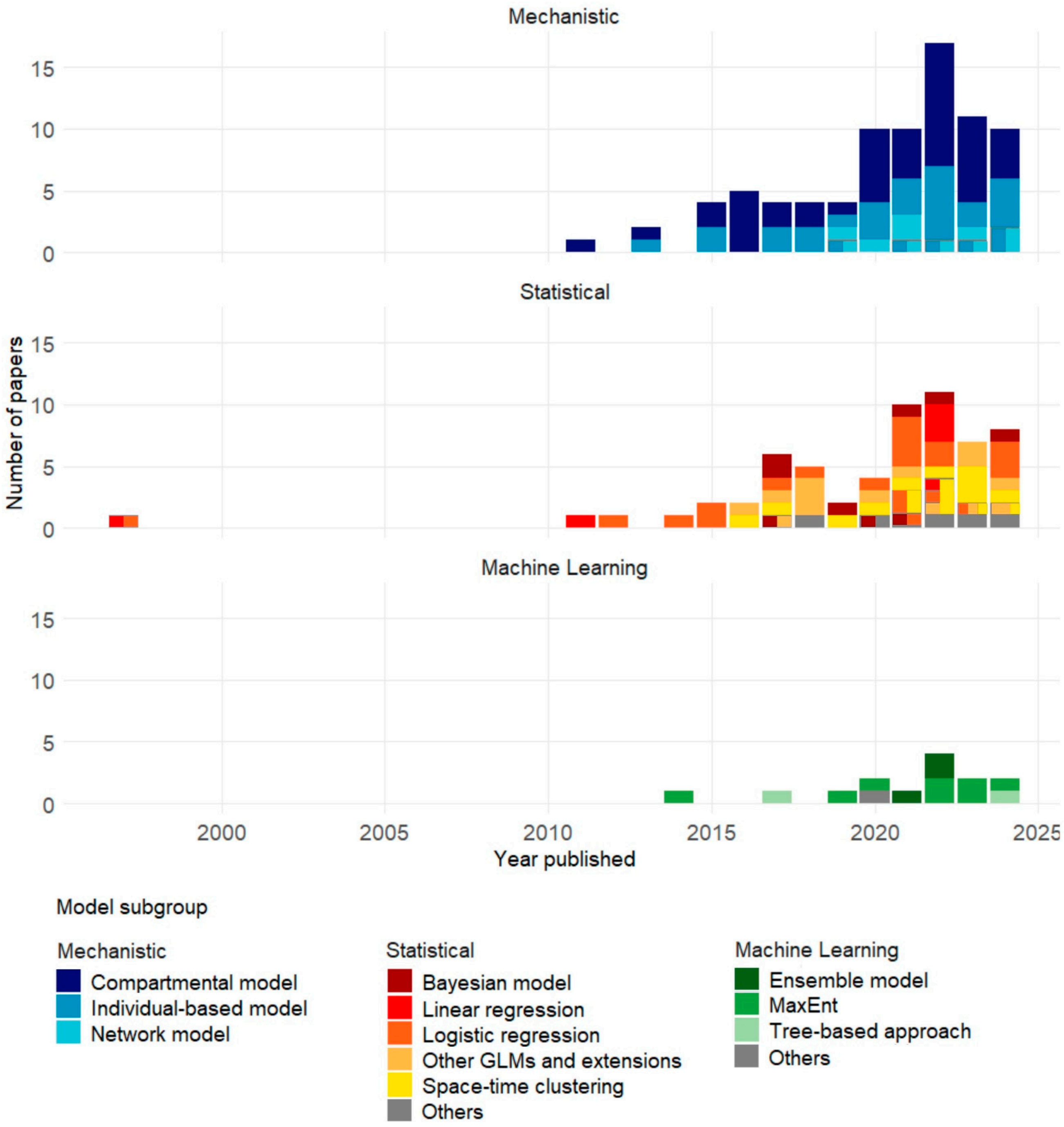

Supplementary Figure S2. Distribution of ASF modelling papers across time and by model subtypes for each modelling approach.

S1E. Estimated basic reproductive number

Supplementary Table S2. Estimated basic reproductive number stratified by host type, geographic context, and model structure.

Reference	R_0 estimate	Transmission Cycle	Host population	Study Location	Model Type	Uncertainty
Mult1 (Gulenkin-2011)	9.8 (3.9-15.6)<br>2-3	Within herd<br>Between herd	Pig	Russian Federation (Europe)	Compartmental model	Deterministic
Mech1 (de Carvalho Ferreira-2013)	18.0 (6.90–46.9)	Within herd	Pig (Malta '78 isolate)	Laboratory (Non-specific)	Compartmental model	Deterministic
Mech3 (Barongo-2015)	1.58 to 3.24 (3.21–3.27) depending on method used	Between herd	Pig	Uganda (Africa)	Compartmental model	Deterministic
Mech6 (Pietschmann-2015)	6.1 (0.5-14.5) or 5.0 (1.4-10.7);<br>0.5 (0.1-1.3)	Within pen<br><br>Between pen	Both	Laboratory (Non-specific)	Compartmental model	Stochastic
Mech8 (Guinat-2016)	2.8 (1.3-4.8)<br>1.4 (0.6, 2.4)	Within pen<br>Between pen	Pig	Laboratory (Non-specific)	Compartmental model	Stochastic
Mech12 (Hu-2017)	24.1 (7.34, 54.2)<br>9.17 (2.67, 19.2)	Within pen<br>Between pen	Pig	Laboratory (Non-specific)	Compartmental model	Stochastic
Mech14 (Nielsen-2017)	1.05 (0.62, 1.72)<br>0.46 (0.17-1.00)	Within pen<br>Between pen	Pig	Laboratory (Non-specific)	Compartmental model	Stochastic
Mech16 (Guinat-2018)	4.4 (2.0-13.4) to 17.3 (3.5-45.5) range from 9 different herds	Within herd	Pig	Russian Federation (Europe)	Compartmental model	Stochastic
Mech26 (Loi-2020)	1.139 (1.123-1.153)<br><br>0.802 (0.612-0.992)	Within nation<br>Basic R0<br>Effective reproduction number	Boar	Italy (Europe)	Compartmental model	Deterministic

Mech45 (Ewing-2022)	0.8 (0.0-2.3) to 12.4 (9.4-16.2) range from 9 different herds	Within herd	Domestic pigs	Russian Federation (Europe)	Individual-based model	Stochastic
Mech49 (Li-2022)	4.82 (3.84, 6.11) to 11.90 (10.71, 12.91) range from 3 different farms	Within herd	Domestic Pigs	China (Asia)	Compartmental model	Deterministic
Mech50 (Mai-2022)	1.49 (1.05–2.21), 1.58 (0.92–2.56), 1.46 (1.38–1.57) depending on method used	Within herd	Domestic Pigs	Vietnam (Asia)	Compartmental model	Deterministic
Mech51 (Mai-2022)	1.41 (1.38–1.44) to 10.8 (10.2–11.1) depending on province	Between herd	Domestic Pigs	Vietnam (Asia)	Compartmental model	Deterministic
Mech64 (Le-2023)	10 (1.1-30)	Within herd	Domestic Pigs	Vietnam (Asia)	Compartmental model	Deterministic
Mech66 (Oh-2023)	2.916 (1.516–7.059) or 4.015 (1.131–9.801) depending on method	Within population	Domestic Pigs	Vietnam (Asia)	Compartmental model	Deterministic

S1E: Estimated basic reproductive number

S1F. Detailed list of methods applied per objective

S1F.1. Mechanistic modelling papers

Supplementary Table S3. Number of mechanistic studies by model framework.

Framework	Compartmental model	Agent-based model	Network model
Population-based			
Space-Yes			
Deterministic	1 (1.1%)	0 (0.0%)	0 (0.0%)
Stochastic	1 (1.1%)	0 (0.0%)	0 (0.0%)
Space-No			
Deterministic	27 (29.7%)	0 (0.0%)	0 (0.0%)
Stochastic	6 (6.6%)	0 (0.0%)	0 (0.0%)
Individual-based			
Space-Yes			
Deterministic	0 (0.0%)	0 (0.0%)	0 (0.0%)
Stochastic	0 (0.0%)	10 (11.0%)	1 (1.1%)
Space-No			
Deterministic	0 (0.0%)	1 (1.1%)	0 (0.0%)
Stochastic	0 (0.0%)	6 (6.6%)	0 (0.0%)
Metapopulation			
Space-Yes			
Deterministic	2 (2.2%)	0 (0.0%)	0 (0.0%)
Stochastic	10 (11.0%)	10 (11.0%)	10 (11.0%)
Space-No			
Deterministic	1 (1.1%)	0 (0.0%)	0 (0.0%)
Stochastic	5 (5.5%)	0 (0.0%)	0 (0.0%)

**Coloured cells indicate non-zero counts.*

Supplementary Table S4. Mechanistic models addressing OBJ 1-Assess consequences of outbreaks.

Framework	Compartmental model	Agent-based model	Network model
Population-based			
Space-Yes			
Deterministic	0 (0.0%)	0 (0.0%)	0 (0.0%)
Stochastic	0 (0.0%)	0 (0.0%)	0 (0.0%)
Space-No			
Deterministic	2 (2.2%)	0 (0.0%)	0 (0.0%)
Stochastic	3 (3.3%)	0 (0.0%)	0 (0.0%)
Individual-based			
Space-Yes			
Deterministic	0 (0.0%)	0 (0.0%)	0 (0.0%)
Stochastic	0 (0.0%)	2 (2.2%)	0 (0.0%)
Space-No			
Deterministic	0 (0.0%)	0 (0.0%)	0 (0.0%)
Stochastic	0 (0.0%)	0 (0.0%)	0 (0.0%)
Metapopulation			
Space-Yes	0 (0.0%)	0 (0.0%)	0 (0.0%)
Deterministic	1 (1.1%)	1 (1.1%)	4 (4.4%)
Stochastic			
Space-No			
Deterministic	0 (0.0%)	0 (0.0%)	0 (0.0%)
Stochastic	0 (0.0%)	0 (0.0%)	0 (0.0%)

**Coloured cells indicate non-zero counts.*

Supplementary Table S5. Mechanistic models addressing OBJ 2-Assess control strategies.

Framework	Compartmental model	Agent-based model	Network model
Population-based			
Space-Yes			
Deterministic	1 (1.1%)	0 (0.0%)	0 (0.0%)
Stochastic	1 (1.1%)	0 (0.0%)	0 (0.0%)
Space-No			
Deterministic	11 (12.1%)	0 (0.0%)	0 (0.0%)
Stochastic	4 (4.4%)	0 (0.0%)	0 (0.0%)
Individual-based			
Space-Yes			
Deterministic	0 (0.0%)	0 (0.0%)	0 (0.0%)
Stochastic	0 (0.0%)	4 (4.4%)	1 (1.1%)
Space-No			
Deterministic	0 (0.0%)	0 (0.0%)	0 (0.0%)
Stochastic	0 (0.0%)	3 (3.3%)	0 (0.0%)
Metapopulation			
Space-Yes			
Deterministic	1 (1.1%)	0 (0.0%)	0 (0.0%)
Stochastic	8 (8.8%)	6 (6.6%)	7 (7.7%)
Space-No			
Deterministic	0 (0.0%)	0 (0.0%)	0 (0.0%)
Stochastic	1 (1.1%)	0 (0.0%)	0 (0.0%)

**Coloured cells indicate non-zero counts.*

Supplementary Table S6. Mechanistic models addressing OBJ 3-Assess model assumptions.

Framework	Compartmental model	Agent-based model	Network model
Population-based			
Space-Yes			
Deterministic	0 (0.0%)	0 (0.0%)	0 (0.0%)
Stochastic	0 (0.0%)	0 (0.0%)	0 (0.0%)
Space-No			
Deterministic	2 (2.2%)	0 (0.0%)	0 (0.0%)
Stochastic	1 (1.1%)	0 (0.0%)	0 (0.0%)
Individual-based			
Space-Yes			
Deterministic	0 (0.0%)	0 (0.0%)	0 (0.0%)
Stochastic	0 (0.0%)	0 (0.0%)	0 (0.0%)
Space-No			
Deterministic	0 (0.0%)	0 (0.0%)	0 (0.0%)
Stochastic	0 (0.0%)	1 (1.1%)	0 (0.0%)
Metapopulation			
Space-Yes			
Deterministic	1 (1.1%)	0 (0.0%)	0 (0.0%)
Stochastic	1 (1.1%)	0 (0.0%)	1 (1.1%)
Space-No			
Deterministic	0 (0.0%)	0 (0.0%)	0 (0.0%)
Stochastic	0 (0.0%)	0 (0.0%)	0 (0.0%)

**Coloured cells indicate non-zero counts.*

S1F: Detailed list of methods applied to address each modelling objective

Supplementary Table S7. Mechanistic models addressing OBJ 4-Assess transmission determinants

Framework	Compartmental model	Agent-based model	Network model
Population-based			
Space-Yes			
Deterministic	0 (0.0%)	0 (0.0%)	0 (0.0%)
Stochastic	0 (0.0%)	0 (0.0%)	0 (0.0%)
Space-No			
Deterministic	12 (13.2%)	0 (0.0%)	0 (0.0%)
Stochastic	2 (2.2%)	0 (0.0%)	0 (0.0%)
Individual-based			
Space-Yes			
Deterministic	0 (0.0%)	0 (0.0%)	0 (0.0%)
Stochastic	0 (0.0%)	6 (6.6%)	1 (1.1%)
Space-No			
Deterministic	0 (0.0%)	0 (0.0%)	0 (0.0%)
Stochastic	0 (0.0%)	3 (3.3%)	0 (0.0%)
Metapopulation			
Space-Yes			
Deterministic	0 (0.0%)	0 (0.0%)	0 (0.0%)
Stochastic	2 (2.2%)	3 (3.3%)	5 (5.5%)
Space-No			
Deterministic	1 (1.1%)	0 (0.0%)	0 (0.0%)
Stochastic	0 (0.0%)	0 (0.0%)	0 (0.0%)

**Coloured cells indicate non-zero counts.*

Supplementary Table S8. Mechanistic models addressing OBJ 5-Estimate parameters.

Framework	Compartmental model	Agent-based model	Network model
Population-based			
Space-Yes			
Deterministic	0 (0.0%)	0 (0.0%)	0 (0.0%)
Stochastic	0 (0.0%)	0 (0.0%)	0 (0.0%)
Space-No			
Deterministic	12 (13.2%)	0 (0.0%)	0 (0.0%)
Stochastic	0 (0.0%)	0 (0.0%)	0 (0.0%)
Individual-based			
Space-Yes			
Deterministic	0 (0.0%)	0 (0.0%)	0 (0.0%)
Stochastic	0 (0.0%)	0 (0.0%)	0 (0.0%)
Space-No			
Deterministic	0 (0.0%)	0 (0.0%)	0 (0.0%)
Stochastic	0 (0.0%)	2 (2.2%)	0 (0.0%)
Metapopulation			
Space-Yes			
Deterministic	0 (0.0%)	0 (0.0%)	0 (0.0%)
Stochastic	1 (1.1%)	2 (2.2%)	1 (1.1%)
Space-No			
Deterministic	0 (0.0%)	0 (0.0%)	0 (0.0%)
Stochastic	4 (4.4%)	0 (0.0%)	0 (0.0%)

**Coloured cells indicate non-zero counts.*

S1F: Detailed list of methods applied to address each modelling objective

Supplementary Table S9. Mechanistic models addressing OBJ 6-Forecast spread.

Framework	Compartmental model	Agent-based model	Network model
Population-based			
Space-Yes			
Deterministic	0 (0.0%)	0 (0.0%)	0 (0.0%)
Stochastic	0 (0.0%)	0 (0.0%)	0 (0.0%)
Space-No			
Deterministic	0 (0.0%)	0 (0.0%)	0 (0.0%)
Stochastic	0 (0.0%)	0 (0.0%)	0 (0.0%)
Individual-based			
Space-Yes			
Deterministic	0 (0.0%)	0 (0.0%)	0 (0.0%)
Stochastic	0 (0.0%)	1 (1.1%)	0 (0.0%)
Space-No			
Deterministic	0 (0.0%)	0 (0.0%)	0 (0.0%)
Stochastic	0 (0.0%)	0 (0.0%)	0 (0.0%)
Metapopulation			
Space-Yes			
Deterministic	1 (1.1%)	0 (0.0%)	0 (0.0%)
Stochastic	4 (4.4%)	0 (0.0%)	1 (1.1%)
Space-No			
Deterministic	0 (0.0%)	0 (0.0%)	0 (0.0%)
Stochastic	0 (0.0%)	0 (0.0%)	0 (0.0%)

**Coloured cells indicate non-zero counts.*

S1F.2. Statistical modelling papers

Supplementary Table S10. Number of papers using specific statistical methods addressed per model subgroup for each modelling objective.

Modelling objective	Model Subtype	Count (%)
Identify risk factors (n = 45)	Logistic regression	**23/45 (51.1%)**
	Logistic regression model	15 (33.3%)
	Conditional logistic regression model	5 (11.1%)
	Generalised Linear Logistic Regression (GLLR)	2 (4.4%)
	Mixed-effects logistic regression model	1 (2.2%)
	Bayesian model	**6/45 (13.3%)**
	Besag, York and Mollie's (BYM) model	3 (6.7%)
	Bayesian space-time model	2 (4.4%)
	Bayesian regression model	1 (2.2%)
	Other GLM extensions	**5/45 (11.1%)**
	Generalised additive model (GAM)	2 (4.4%)
	Generalised linear mixed models (GLMM) with binary response (logit link)	2 (4.4%)
	GLMM with Poisson distribution	1 (2.2%)
	Negative binomial regression	**4/45 (8.9%)**
	Poisson regression	**4/45 (8.9%)**
	Conditional autoregressive zero-inflated Poisson regression (CAR-ZIP)	1 (2.2%)
	Mixed-effects Poisson regression model	1 (2.2%)
	Poisson regression model	1 (2.2%)
	Spatial zero-inflated Poisson regression model	1 (2.2%)
	Linear regression	**3/45 (6.7%)**
Identify spatial and temporal patterns (n = 21)	Space-time clustering	**17/21 (81.0%)**
	Kulldorff space-time permutation scan statistic	17 (81.0%)
	Spatial model	**2/21 (9.5%)**
	Kriging model with semi-variogram	2 (9.5%)

	Bayesian model	**1/21 (4.8%)**
	Bayesian space-time model	1 (4.8%)
	Linear regression	**1/21 (4.8%)**
Forecast spread (n = 5)	Poisson regression	**2/5 (40.0%)**
	Poisson regression model	1 (20.0%)
	Zero-inflated Poisson regression model	1 (20.0%)
	Logistic model	**1/5 (20.0%)**
		1 (20.0%)
	Negative binomial regression	**1/5 (20.0%)**
		1 (20.0%)
	Other GLM extensions	**1/5 (20.0%)**
	Generalised additive model (GAM)	1 (20.0%)
Estimate outbreak risk (n = 2)	Risk assessment model	**1/2 (50.0%)**
	Binomial likelihood function	1 (50.0%)
	Survival model	**1/2 (50.0%)**
	Cox regression model	1 (50.0%)
Identify predictors (n = 2)	Linear regression	**1/2 (50.0%)**
	Other GLM extensions	**1/2 (50.0%)**
	Generalised linear mixed-effects model	
Identify temporal trends (n = 2)	Bayesian model	**1/2 (50.0%)**
	Linear regression	**1/2 (50.0%)**
Assess control strategies (n = 1)	Bayesian model	**1/1 (100.0%)**
	Bayesian regression model	1 (100.0%)

S1F.3. Machine Learning modelling papers

Supplementary Table S11. Number of papers using specific machine learning methods addressed per model subgroup for each modelling objective.

Modelling objective	Model Subtype	Count (%)
Estimate suitability of disease occurrence; Identify predictors	Maximum entropy modelling	8/9 (88.9)%
	Ensemble modelling	1/9 (11.1)%
Identify risk factors	Tree-based approach	4/4 (100.0)%
	Random Forest (RF)	2 (50%)
	Classification Tree Analysis (CTA)	1 (25%)
	Boosted Regression Tree (BRT) model	1 (25%)
Identify spatial and temporal patterns	Space-time clustering	1/1 (100.0)%

S1G. Model adequacy and uncertainty quantification by approach

Supplementary Table S12. Summary for mechanistic models.

Dimension and values	Count	Proportion (%)
(1) Uncertainty for model output (n = 66)		
Simulation	31	47.0%
Bayesian	4	6.1%
Not reported	31	47.0%
(2) Uncertainty for estimated parameters (n = 22)		
Frequentist	12	54.5%
Bayesian	10	45.5%
Simulation	2	9.1%
(3) Sensitivity Analysis (n = 82)		
Model parameters	38	46.3%
Model assumptions	3	3.7%
Mechanistic assumptions	2	2.4%
Initial conditions	1	1.2%
Not performed	39	47.6%
No uncertainty quantification in any of 3 categories	12	14.6%

Supplementary Table S13. Summary for statistical models.

Dimension and values	Count	Proportion (%)
(1) Model Adequacy Checks (n = 61)		
Not reported	30	49.2%
AIC	13	21.3%
AUC-ROC	8	13.1%
Hosmer and Lemeshow GOF test	5	8.2%
Likelihood ratio test	5	8.2%
R-squared	5	8.2%
Deviance information criteria (DIC)	3	4.9%
BIC	2	3.3%
RMSE	2	3.3%
Concordance	1	1.6%
Convergence	1	1.6%
Effective number of parameters (pD)	1	1.6%
Le Cessie-van Houwelingen normal test	1	1.6%
MAPE	1	1.6%
Minimum convex polygon ratio	1	1.6%
Pearson GOF test	1	1.6%
Residuals analysis	1	1.6%
(2) Assumptions Checking (n = 61)		
Not reported	31	50.8%
Multicollinearity	13	21.3%
Spatial autocorrelation	10	16.4%
Normality	5	8.2%
Overdispersion	3	4.9%
Correlation	2	3.3%
Assumption of proportional hazards	1	1.6%
Confounding	1	1.6%
Convergence of sampling for posterior distribution	1	1.6%
Handling ties	1	1.6%
Heteroscedasticity	1	1.6%
Seasonality test	1	1.6%
(3) Model Selection (n = 47)		
All considered variables	15	31.9%

Stepwise backward selection	11	23.4%
Stepwise bidirectional selection	6	12.8%
Stepwise forward selection	6	12.8%
Univariable analysis where P<alpha	6	12.8%
Combination of variables with lowest AIC	2	4.3%
Features where P<alpha	1	2.1%

Supplementary Table S14. Summary for machine leaning.

**Dimension and values**	**Count**	**Proportion (%)**
(1) Model Adequacy Checks (n = 14)		
AUC-ROC	12	85.7%
True skill statistic	5	35.7%
Cohen's Kappa	3	21.4%
Sensitivity	2	14.3%
Accuracy	1	7.1%
Matthews Correlation Coefficient	1	7.1%
Specificity	1	7.1%
(2) Assumptions Checking (n = 14)		
Multicollinearity	9	64.3%
Not reported	5	35.7%
Spatial autocorrelation	1	7.1%
Low variance	1	7.1%
(3) Model Selection (n = 14)		
Assumptions checking	6	42.9%
Minimum model contribution	5	35.7%
All considered variables	3	21.4%
CFS optimal subset of features	1	7.1%
Stepwise backward selection	1	7.1%
(4) Feature importance/Interpretability tools (n = 14)		
Jackknife test	6	42.9%
Relative contribution	4	28.6%
Permutation importance	2	14.3%
Variable response curves	2	14.3%
AUC-ROC	1	7.1%
Cross-validation error	1	7.1%
Logistic regression	1	7.1%
Mean contribution	1	7.1%
Out-of-bag (OOB)	1	7.1%
Variable importance based on pruned tree	1	7.1%
(5) Data Splitting (n = 14)		
Two-way split	11	78.6%
Cross-validation	2	14.3%
Out-of-bag (bootstrapping)	1	7.1%
(6) Uncertainty Quantification (n = 14)		
Not reported	9	64.3%
Simulation/sampling based	3	21.4%
Frequentist	1	7.1%
Mask clamping	1	7.1%

S1G: Model adequacy and uncertainty quantification by approach

S1H. Clustering analysis using HCPC

R code for Hierarchical Clustering on Principal Components (HCPC) analysis

```
library(FactoMineR)
library(dplyr)
library(tidyverse)

# Load HCPC dataset
# Expected columns: ModelImpID, Year (numeric), Continent, Approach, Objective
df <- read_csv("papers-by-approach-year-loc-obj.csv")
df <- df %>% select(ModelImpID, Year, Continent, Approach, Objective) %>%
  as.data.frame() %>%
  mutate(Year = as.numeric(Year), across(c(Continent, Approach, Objective),
as.factor))

# Each row represents a model implementation
df_hcpc <- df %>% select(-ModelImpID)
rownames(df_hcpc) <- df$ ModelImpID

# Run FAMD and HCPC
set.seed(1)
famd <- FAMD(df_hcpc, graph = FALSE)
hc   <- HCPC(famd, nb.clust = -1, consol = TRUE, graph = FALSE)

# Extract cluster assignments
assignments <- hc$data.clust %>% as_tibble(rownames = "ModelImpID") %>%
  transmute(ModelImpID, cluster = factor(clust)) %>% mutate(ModelImpID =
as.numeric(ModelImpID))
df_clusters <- df %>% left_join(assignments, by = "PaperID")

# Interpret clusters
cat_list <- hc$desc.var$category
quanti_list <- hc$desc.var$quanti # Result for ‘Year’ is NULL

# Supplementary analysis: 'Year' summary and Kruskal-Wallis test
year_by_cluster <- df_clusters %>%
  group_by(cluster) %>%
  summarise(
    n = n(),
    mean_year   = mean(Year, na.rm = TRUE),
    sd_year     = sd(Year, na.rm = TRUE),
    median_year = median(Year, na.rm = TRUE),
    iqr_year    = IQR(Year, na.rm = TRUE)
  )
dat <- df_clusters %>%
  filter(!is.na(Year), !is.na(cluster)) %>%
  mutate(cluster = as.factor(cluster),
         Year = as.numeric(Year))
kw <- kruskal.test(Year ~ cluster, data = dat)
```

Hierarchical Clustering on Principal Components (HCPC) results

For each cluster, category levels with statistically significant positive v-tests are reported, indicating over-representation relative to the dataset overall (Supplementary Table 12), and levels with significant negative v-tests, indicating under-representation (Supplementary Table 13). Columns show Mod/Cla (percentage of the cluster that has that category level), Cla/Mod (percentage of the level that falls in the cluster), Global prevalence, v-test (standardised enrichment statistic), and p-value. Statistical significance is evaluated at $\alpha = 0.05$. These results are generated using Hierarchical Clustering on Principle Components (HCPC) using 'FactoMineR' package in R version 4.4.0, applying Factor Analysis of Mixed Data (FAMD) and the number of clusters determined by the automated HCPC criterion.

Supplementary Table S15. Category enrichment per cluster (over-represented levels).

Cluster	Category	Level	Mod/Cla (%)	Cla/Mod (%)	Global (%)	v-test	p-value
1	Approach	Mechanistic	100	100	53	16.9	<0.001
1	Objective	Assess control strategies	97.6	35.1	19.1	6.9	<0.001
1	Objective	Assess transmission determinants	100	29.8	15.8	6.7	<0.001
1	Objective	Estimate parameters	100	19.3	10.2	5.1	<0.001
1	Objective	Assess consequences of outbreaks	100	7.9	4.2	3	0.003
1	Objective	Assess model assumptions	100	4.4	2.3	2.1	0.04
1	Continent	Not specified	100	26.3	14	6.2	<0.001
1	Continent	Americas	93.3	12.3	7	3.4	<0.001
2	Approach	Statistical	100	95.1	36.3	15.5	<0.001
2	Objective	Identify risk factors	98	58.5	22.8	10.2	<0.001
2	Objective	Identify spatial and temporal patterns	100	26.8	10.2	6.5	<0.001
2	Continent	Europe	54.3	61	42.8	4.2	<0.001
2	Continent	Africa	72.7	9.8	5.1	2.3	0.022
3	Approach	Machine Learning	82.6	100	10.7	10	<0.001
3	Objective	Estimate suitability of disease occurrence	100	47.4	4.2	6.6	<0.001
3	Objective	Identify predictors	81.8	47.4	5.1	6	<0.001
3	Continent	Asia	23	73.7	28.4	4.2	<0.001
3	Continent	Global	100	21.1	1.9	4.1	<0.001

Supplementary Table S16. Category depletion per cluster (under-represented levels).

Cluster	Category	Level	Mod/Cla (%)	Cla/Mod (%)	Global (%)	v-test	p-value
1	Continent	World	0	0	1.9	-2	0.047
1	Continent	Europe	44.6	36	42.8	-2.1	0.033
1	Continent	Asia	39.3	21.1	28.4	-2.5	0.012
1	Objective	Estimate suitability of disease occurrence	0	0	4.2	-3.3	<0.001
1	Objective	Identify predictors	0	0	5.1	-3.7	<0.001
1	Objective	Identify spatial and temporal patterns	0	0	10.2	-5.7	<0.001
1	Approach	Machine Learning	0	0	10.7	-5.8	<0.001
1	Objective	Identify risk factors	0	0	22.8	-9.2	<0.001
1	Approach	Statistical	0	0	36.3	-12.9	<0.001
2	Approach	Machine Learning	17.4	4.9	10.7	-2.2	0.028

2	Objective	Estimate suitability of disease occurrence	0	0	4.2	-2.5	0.012
2	Objective	Assess consequences of outbreaks	0	0	4.2	-2.5	0.012
2	Continent	Americas	6.7	1.2	7	-2.7	0.007
2	Objective	Estimate parameters	0	0	10.2	-4.4	<0.001
2	Continent	Simulated/Non-specific settings	0	0	14	-5.3	<0.001
2	Objective	Assess transmission determinants	0	0	15.8	-5.7	<0.001
2	Objective	Assess control strategies	2.4	1.2	19.1	-5.8	<0.001
2	Approach	Mechanistic	0	0	53	-13.5	<0.001
3	Objective	Identify risk factors	2	5.3	22.8	-2	0.047
3	Objective	Assess transmission determinants	0	0	15.8	-2.1	0.032
3	Objective	Assess control strategies	0	0	19.1	-2.4	0.015
3	Continent	Europe	1.1	5.3	42.8	-3.7	<0.001
3	Approach	Statistical	0	0	36.3	-3.9	<0.001
3	Approach	Mechanistic	0	0	53	-5.2	<0.001

S1H: Clustering analysis using HCPC
